# Decoding molecular heterogeneity in lupus nephritis: integrating single-cell and spatial multi-omics for endotype-driven precision therapeutics

**DOI:** 10.3389/fimmu.2026.1987126

**Published:** 2026-10-14

**Authors:** Jun Zhou, Wei Xiao, Hui Jian

**Affiliations:** 1 Department of Traditional Chinese Medicine, Jiangxi University of Chinese Medicine, Nanchang, Jiangxi, China; 2 Department of Traditional Chinese Medicine, Gaoxin Branch of the First Affiliated Hospital, Nanchang University, Nanchang, Jiangxi, China

**Keywords:** lupus nephritis, molecular disease heterogeneity, precision renal medicine, single-cell transcriptomics, spatial multi-omics, urinary biomarkers

## Abstract

Lupus nephritis (LN) develops in 40–60% of patients with systemic lupus erythematosus (SLE) and remains the leading cause of renal morbidity and progression to end-stage renal disease (ESRD). While the upstream immunological events initiating LN—including loss of self-tolerance, pathogenic autoantibody production, glomerular immune complex deposition, and type I interferon (IFN) activation—are broadly shared, accumulating evidence demonstrates that these convergent triggers give rise to molecularly heterogeneous tissue injury programs at the renal level. This fundamental divergence provides a mechanistic explanation for the inconsistent therapeutic responses observed with standardized immunosuppressive protocols. Advances in single-cell transcriptomics, spatial gene expression mapping, CITE-seq, and cross-platform multi-omic integration have deconvolved this heterogeneity into at least five recognizable tissue-level injury programs: (i) an IFN-γ-polarized macrophage–tubular epithelial cell co-activation circuit, (ii) a Tfh–B cell ectopic germinal center pathway, (iii) a neutrophil extracellular trap (NET)-driven inflammatory cascade, (iv) a podocyte-autonomous injury program, and (v) a TREM2+ macrophage-orchestrated fibrotic program. Concurrently, multiplexed urinary biomarker platforms are under development as non-invasive surrogates for tissue-level pathogenic activity. This review synthesizes current understanding of disease mechanisms, molecular profiling evidence, biomarker candidates, and mechanism-directed therapies corresponding to each tissue injury program. We map existing and investigational agents—including anifrolumab, obinutuzumab, voclosporin, CAR-T cell therapy, and complement inhibitors—to their mechanistic targets and delineate the design requirements for prospective biomarker-stratified trials essential to validating molecularly guided treatment selection.

## 1 Introduction

Systemic lupus erythematosus (SLE) stands as the archetypal multi-organ autoimmune condition, characterized by the generation of antibodies directed against chromatin and other nuclear constituents, deposition of the resulting immune complexes across susceptible tissues, and consequent inflammatory organ damage (1). The kidney is among the most frequently and severely affected organs: lupus nephritis (LN) develops in roughly 40–60% of individuals with SLE and remains the single strongest predictor of adverse long-term patient outcomes, including premature cardiovascular mortality and accelerated loss of renal function (2, 3). Despite the availability of modern combination immunosuppressive regimens, patients with proliferative forms of LN face a 5–10-fold excess risk of progressing to end-stage renal disease (ESRD) compared to age-matched controls, and approximately 10–30% reach ESRD within ten years of their initial nephritis diagnosis (4, 5).

The established pathogenic sequence in LN follows a well-defined trajectory: defective clearance of apoptotic cellular debris results in sustained exposure of nuclear autoantigens to the adaptive immune system; cognate T cell help drives B cell affinity maturation within germinal centers, yielding high-affinity anti-dsDNA and anti-nucleosome autoantibodies; these autoantibodies assemble into immune complexes that become trapped in glomerular capillary walls and mesangium; and local engagement of complement receptors and Fcγ receptors triggers robust recruitment of myeloid and lymphoid effector populations into the renal interstitium (6, 7). While this sequential model remains foundational, it cannot adequately explain the remarkable heterogeneity in clinical behavior observed among patients who share identical histopathological classifications. The emergence of single-cell resolution sequencing technologies over the past half-decade has revealed extensive molecular diversity within both the intrarenal and peripheral immune compartments of LN patients that conventional morphological assessment fails to detect (8, 9).

Contemporary clinical management of LN is anchored to the ISN/RPS histological classification framework—updated in 2018 to improve lesion definitions and activity/chronicity scoring—which stratifies disease from Class I (minimal mesangial deposits) through Class VI (globally sclerotic disease) and directly informs treatment selection (10, 11). Nevertheless, widely acknowledged limitations of this morphology-centered system persist: histological class correlates only loosely with underlying molecular pathogenic activity; sequential biopsies commonly reveal unexpected transitions between classes; and individuals assigned to the same histological category frequently harbor distinct transcriptomic programs and follow markedly divergent clinical courses (12). These recognized deficiencies underscore a pressing requirement for molecular-level stratification tools capable of supplementing—and, in appropriate clinical contexts, potentially superseding—traditional histopathological evaluation.

**Table 1** provides a comprehensive summary of the ISN/RPS histological classification system, including the defining pathological features, predominant immune complex deposition patterns, typical clinical presentations, and recommended therapeutic approaches for each class. Importantly, it must be noted that many LN biopsies demonstrate mixed histological classifications (e.g., Class III+V or Class IV+V), occurring in approximately 15–30% of cases depending on the cohort. These mixed-class presentations often exhibit clinical and prognostic features intermediate between the component classes. Furthermore, the histological class identified in a given biopsy is inherently dependent on the specific location within the kidney cortex from which the biopsy core is procured: focal lesions (Class III) may be missed if the biopsy needle does not traverse affected glomeruli, and the proportion of globally sclerosed glomeruli varies substantially between cortical regions. A minimum of 10 glomeruli is generally recommended for reliable classification, though even this threshold does not eliminate sampling variability (10, 11, 13, 14).The classical understanding of LN immunopathogenesis, encompassing the key pathogenic components from loss of self-tolerance through tissue injury and fibrosis, is summarized in **Figure 1**.

**Table 1:** ISN/RPS histological classification of lupus nephritis.

ISN/RPS class	Histological designation	Key pathological features	Immune complex deposition pattern	Clinical presentation	Therapeutic approach	Notes
Class I	Minimal mesangial LN	Normal light microscopy; mesangial immune deposits visible only on IF/EM	Mesangial deposits (IF/EM only)	Usually asymptomatic; normal urinalysis or mild hematuria	Monitor; treat extrarenal SLE	May progress to higher classes; requires serial monitoring
Class II	Mesangial proliferative LN	Mesangial hypercellularity with mesangial matrix expansion; no endocapillary or extracapillary proliferation	Mesangial deposits (visible on LM, IF, EM)	Mild proteinuria (<1 g/day); microscopic hematuria	Conservative management; low-dose immunosuppression if progressive proteinuria	Transition to Class III/IV possible; monitor renal function
Class III	Focal LN (III-A, III-A/C, III-C)	Active and/or chronic glomerular lesions in <50% of glomeruli; endocapillary hypercellularity ± crescents	Subendothelial deposits (focal distribution); mesangial deposits	Proteinuria (often >1 g/day); hematuria; variable renal function impairment	Immunosuppressive induction: MMF or low-dose CYC + glucocorticoids	Subclassified by activity (A) and chronicity (C); may coexist with Class V
Class IV	Diffuse LN (IV-S/IV-G, IV-A, IV-A/C, IV-C)	Active and/or chronic glomerular lesions in ≥50% of glomeruli; subendothelial deposits ± crescents; wire-loop lesions	Subendothelial deposits (diffuse); mesangial deposits; ± subepithelial deposits	Nephritic syndrome; hypertension; renal insufficiency; possible rapid GFR decline	Aggressive induction: MMF or CYC + glucocorticoids; consider multi-target therapy	Most severe proliferative form; segmental (S) vs global (G); highest risk for ESRD
Class V	Membranous LN	Diffuse subepithelial immune deposits; GBM thickening; may have mesangial proliferation	Subepithelial deposits; GBM thickening on EM	Nephrotic syndrome (proteinuria >3.5 g/day); edema; hyperlipidemia; thrombotic risk	Immunosuppression (MMF, CNI, or CYC) ± anticoagulation for thrombotic risk	May occur in pure form or combined with Class III or IV (mixed-class)
Class VI	Advanced sclerotic LN	≥90% globally sclerosed glomeruli; advanced interstitial fibrosis and tubular atrophy	Variable; often obscured by sclerosis	Chronic renal failure; irreversible kidney damage	Renal replacement therapy (dialysis or transplantation); treat extrarenal SLE	End-stage; immunosuppression no longer effective for renal recovery

¹Mixed-class presentations (e.g., Class III+V, Class IV+V) occur in approximately 15–30% of biopsies and exhibit clinical features intermediate between component classes. ²The histological class identified is dependent on the specific cortical region sampled; focal lesions may be missed if the biopsy does not traverse affected glomeruli. ³A minimum of 10 glomeruli is recommended for reliable classification, though sampling variability cannot be entirely eliminated (10, 11, 13, 14).

ISN/RPS, International Society of Nephrology/Renal Pathology Society; LM, light microscopy; IF, immunofluorescence; EM, electron microscopy; GBM, glomerular basement membrane; MMF, mycophenolate mofetil; CYC, cyclophosphamide; CNI, calcineurin inhibitor; ESRD, end-stage renal disease.

**Figure 1:**
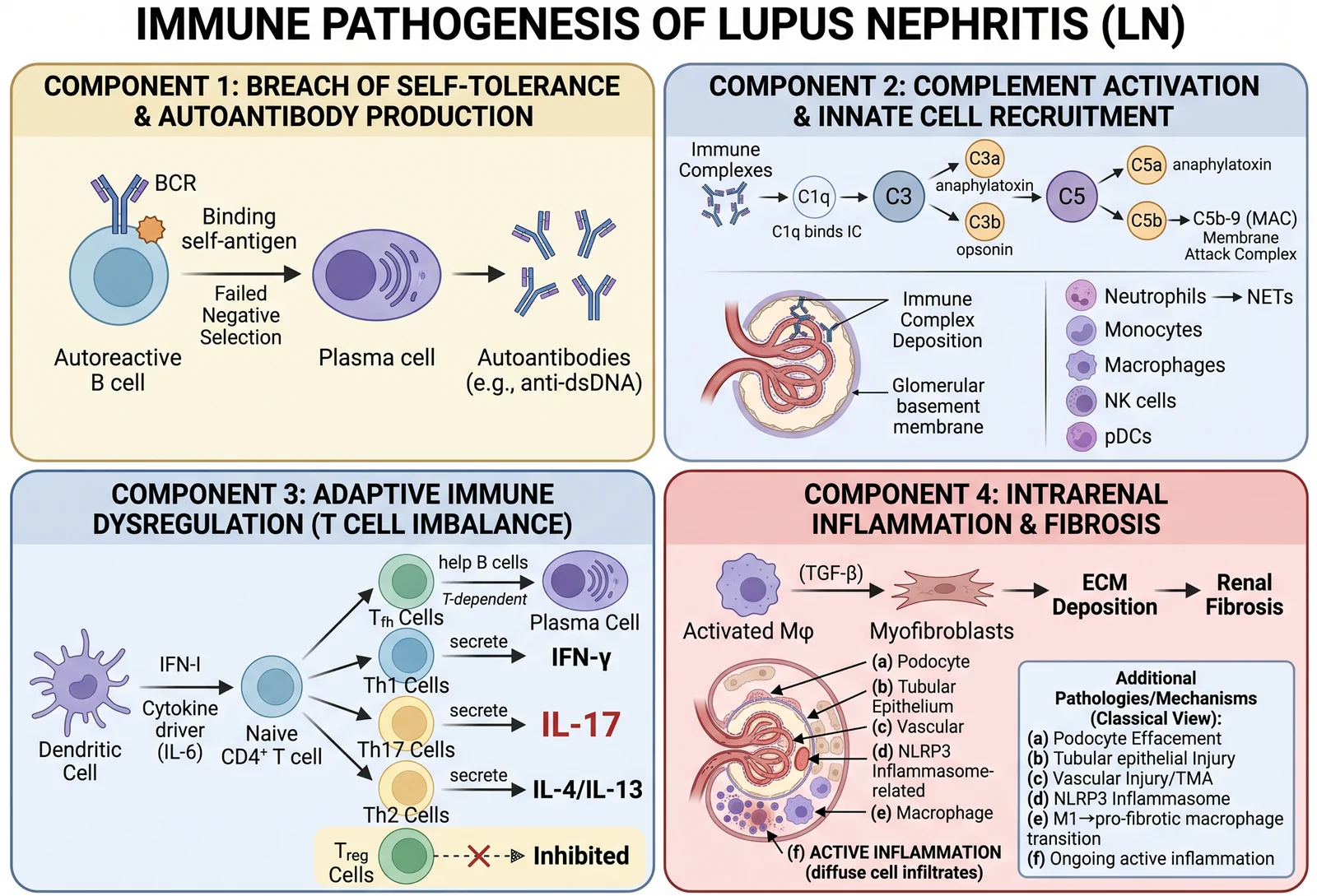
Classical understanding of lupus nephritis immunopathogenesis (pre-multi-omic framework). Schematic overview of the classical understanding of LN immunopathogenesis, organized by overlapping pathogenic components. Component 1: Breach of self-tolerance and autoantibody production via failed negative selection and T cell-dependent germinal center reactions. Component 2: Complement activation (with C3 as the central convergence point) and innate cell recruitment, including neutrophils, monocytes, macrophages, NK cells, and pDCs. Component 3: Adaptive immune dysregulation (T cell imbalance), with expansion of Th1, Th2, Th17, and Tfh subsets, concurrent Treg impairment, and IFN-gamma/IL-17 production. Component 4: Intrarenal inflammation and fibrosis, including TGF-beta-driven macrophage-to-myofibroblast transition, ECM deposition, and additional pathologies including podocyte effacement, tubular epithelial injury, vascular injury/TMA, NLRP3 inflammasome activation, and M1-to-pro-fibrotic macrophage transition. This image summarizes the classical understanding; novel findings from recent multi-omic studies are summarized in **Figure 2**.

**Figure 2:**
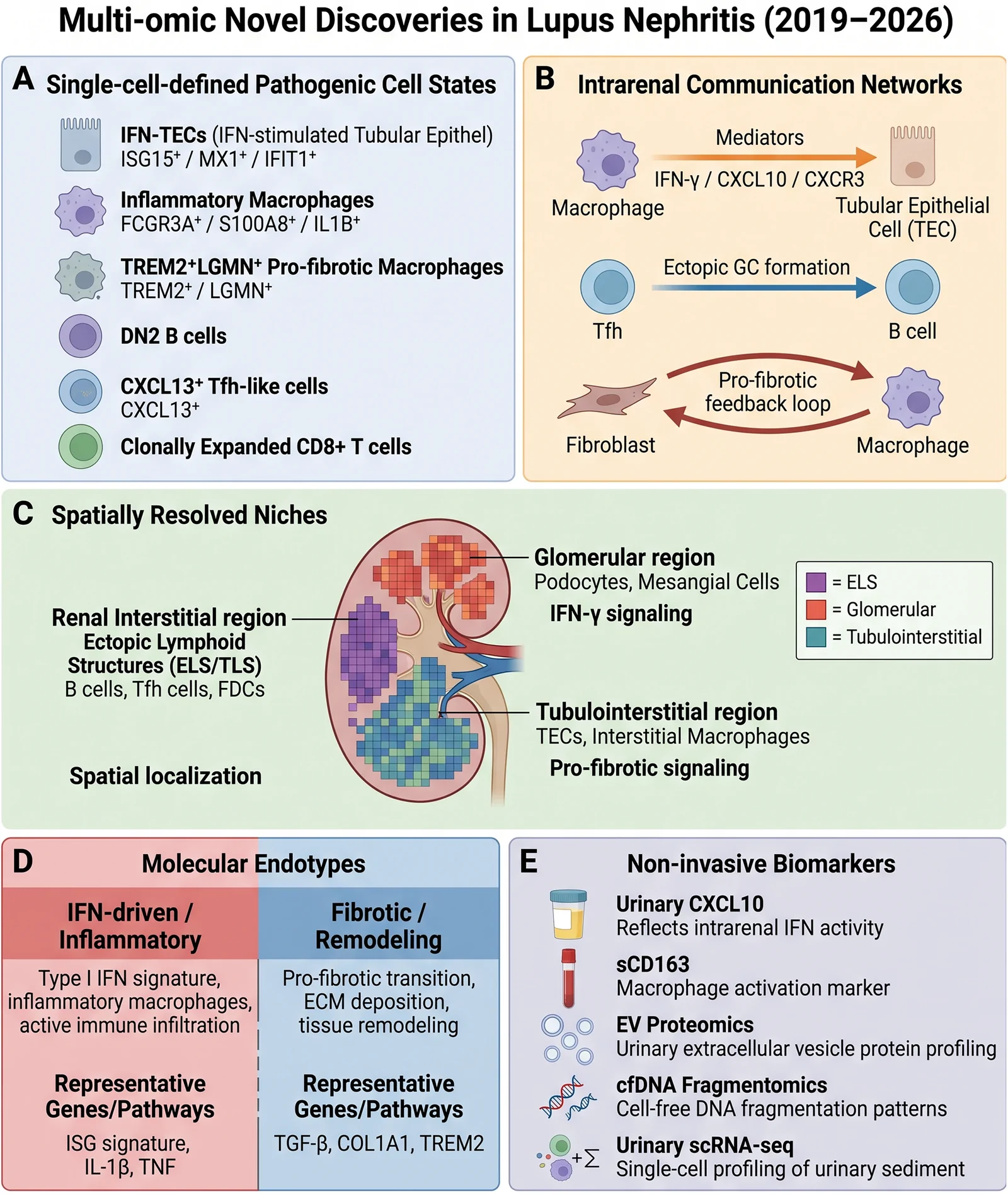
Multi-omic novel discoveries in lupus nephritis (2019–2026). Summary of key novel findings from single-cell, spatial, and multi-omic studies of LN. **(A)** Single-cell-defined pathogenic cell states: IFN-TECs (ISG15+/MX1+/IFIT1+); inflammatory macrophages (FCGR3A+/S100A8+/IL1B+); TREM2+LGMN+ pro-fibrotic macrophages; DN2 B cells; CXCL13+ Tfh-like cells; clonally expanded CD8+ T cells. **(B)** Intrarenal communication networks: macrophage-TEC niche via IFN-gamma/CXCL10/CXCR3; ectopic germinal center formation; fibroblast-macrophage pro-fibrotic feedback loop. **(C)** Spatially resolved niches: tertiary lymphoid structures (TLS) in the renal interstitial region; IFN-gamma signaling in the glomerular region; pro-fibrotic signaling in the tubulointerstitial region. **(D)** Molecular endotypes: IFN-driven/inflammatory (ISG signature, IL-1beta, TNF) vs. fibrotic/remodeling (TGF-beta, COL1A1, TREM2). **(E)** Non-invasive biomarkers: urinary CXCL10, sCD163, EV proteomics, cfDNA fragmentomics, and urinary scRNA-seq.

The central organizing principle of this review can be stated concisely: LN follows a convergent-to-divergent pathogenic trajectory. At the systemic level, a shared set of upstream triggers—breach of immunological self-tolerance, generation of anti-nuclear autoantibodies, glomerular immune complex accumulation, and activation of type I IFN signaling—are common across virtually all patients. At the tissue level, however, these convergent inputs give rise to molecularly heterogeneous disease programs that differ in their predominant immune effector populations, dominant cytokine signaling axes, and mechanisms of parenchymal injury. This molecular divergence at the point of tissue engagement offers a compelling explanation for why uniform immunosuppressive regimens—designed to target convergent upstream processes—achieve only partial efficacy in many patients: such regimens may leave the specific divergent pathogenic pathway driving an individual patient’s renal injury inadequately addressed. The convergent-to-divergent framework, depicted schematically in **Figure 3**, therefore furnishes both the scientific rationale for molecular endotyping and the clinical logic for pathway-targeted precision therapeutics in LN (8, 15, 16).

**Figure 3:**
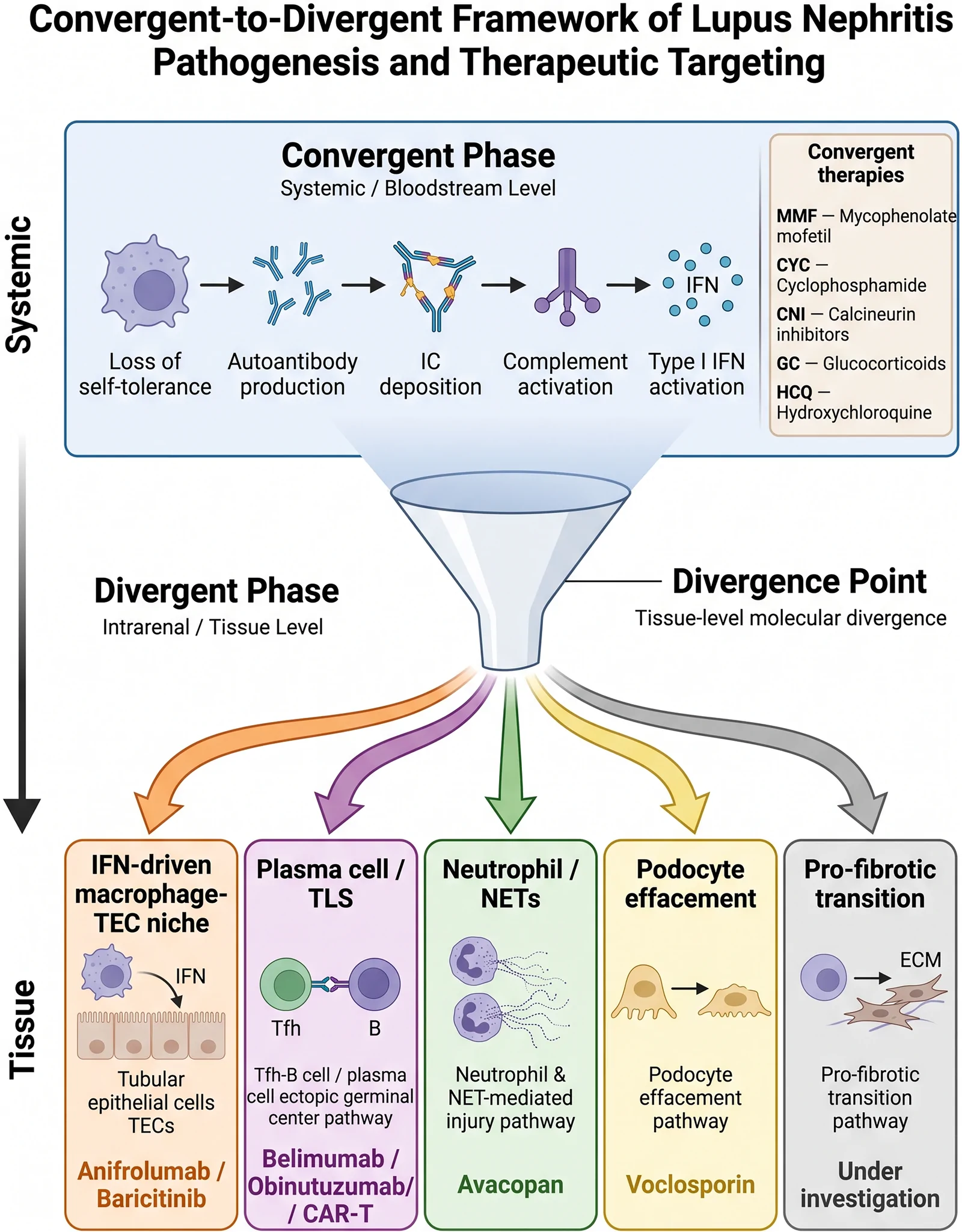
Convergent-to-divergent framework of lupus nephritis pathogenesis and therapeutic targeting. Schematic overview illustrating the convergent-to-divergent biological trajectory of lupus nephritis. The upper portion depicts convergent systemic triggers (loss of self-tolerance, autoantibody production, immune complex deposition, complement activation, type I IFN activation) shared across patients. Below the divergence point, the disease branches into molecularly distinct tissue-level pathways: (i) IFN-driven macrophage-TEC inflammatory niche; (ii) Tfh-B cell/plasma cell ectopic germinal center pathway with tertiary lymphoid structures; (iii) neutrophil/NET-mediated injury pathway; (iv) podocyte effacement and lupus podocytopathy pathway; (v) pro-fibrotic transition pathway. Current convergent-targeting therapies and divergent-targeting therapies are mapped to their respective target locations. MMF, mycophenolate mofetil; CYC, cyclophosphamide; CNI, calcineurin inhibitors; GC, glucocorticoids; HCQ, hydroxychloroquine; TEC, tubular epithelial cell; TLS, tertiary lymphoid structure; NETs, neutrophil extracellular traps; ECM, extracellular matrix; IFN, interferon.

Multi-layered molecular profiling strategies—spanning genomics, transcriptomics, proteomics, metabolomics, and epigenomics—provide a systematic means of interrogating LN biology across multiple interconnected biological scales. The Accelerating Medicines Partnership (AMP) consortium has assembled single-cell transcriptomic and surface protein profiles from renal biopsy tissue and matched peripheral blood specimens from large cohorts of LN patients, generating the most detailed molecular landscape of the disease currently available (8). These comprehensive datasets have uncovered cell type-resolved gene expression modules linked to histological classification and clinical trajectory, yielding candidate molecular markers and potential therapeutic targets now entering prospective evaluation. Importantly, such integrated multiomics analytical frameworks are not unique to LN; analogous approaches incorporating genomic, transcriptomic, and proteomic data have been productively applied across diverse disease contexts, including pan-cancer analyses that have identified key post-transcriptional regulators such as ELAVL1 through cross-platform data integration (17).

Guided by this pathogenesis-to-therapy analytical framework, this review systematically examines three interconnected questions central to the advancement of precision renal medicine in LN: (i) Which immune cell populations, cytokine networks, and intracellular signaling cascades represent shared upstream pathogenic triggers, and which constitute pathway-specific tissue-level injury programs? (ii) How have single-cell, spatially resolved, and integrated multi-omic profiling technologies mapped the landscape of molecular disease heterogeneity, and what candidate biomarker panels and disease subtype classifications have emerged from these studies? (iii) Which existing and investigational therapeutic agents are directed at shared versus pathway-specific disease mechanisms, and what clinical trial designs are needed to rigorously validate molecularly guided treatment allocation? Our analysis emphasizes evidence generated within the past five years, giving particular weight to studies employing single-cell resolution or spatially contextualized profiling.

This review is intentionally organized not as a platform-by-platform technology survey—the structural approach adopted by most prior reviews of LN molecular profiling (16)—but rather employs the shared-to-branching pathogenic model as its central analytical framework. By structuring the evidence around common upstream mechanisms versus pathway-specific tissue programs, we seek to offer both clinicians and laboratory investigators a coherent framework that directly links molecular findings to therapeutic decision-making. This organizational principle conveys a substantive clinical message: the well-documented therapeutic failure of standardized immunosuppressive regimens in a sizeable proportion of LN patients may not indicate inadequate pharmacological potency, but instead may reflect a fundamental mismatch between treatments designed to suppress shared upstream processes and the pathway-specific molecular diseases actually operative at the tissue level. Throughout this review, we rigorously evaluate where multi-omic data support genuinely reproducible molecular disease subtypes versus where apparent heterogeneity may reflect methodological inconsistency between studies, and we clearly delineate the translational distance between exploratory molecular profiling and the analytically validated, clinically grade-ready tools needed before molecular classifications can appropriately guide patient management.

## 2 Shared upstream pathogenic mechanisms in lupus nephritis

### 2.1 Breakdown of self-tolerance and generation of nephritogenic autoantibodies

The pathogenic sequence culminating in LN begins with breakdown of both central and peripheral immunological tolerance, encompassing defective thymic negative selection of self-reactive T cell clones (18) and impaired peripheral clearance of apoptotic cell remnants attributable to complement component deficiencies and diminished DNase I enzymatic activity (19, 20). Plasmacytoid dendritic cells (pDCs) encountering nucleic acid-containing immune complexes elaborate type I interferons (IFN-I), which reduce the activation threshold for self-reactive lymphocytes (21, 22). Self-reactive B cells subsequently undergo somatic hypermutation and affinity maturation within germinal centers, producing high-affinity anti-dsDNA, anti-nucleosome, and anti-C1q autoantibodies that assemble into pathogenic immune complexes and become deposited within glomerular structures (23). The anatomical location of immune complex deposition largely dictates the resulting histopathological pattern: mesangial localization corresponds to Class II, subendothelial deposits produce the proliferative lesions of Class III/IV, and subepithelial deposits are characteristic of membranous Class V LN (10).

### 2.2 Complement pathway activation and mobilization of innate immune effectors

Deposition of immune complexes within glomerular structures triggers classical complement pathway activation through C1q recognition, generating the anaphylatoxins C3a and C5a that serve as potent chemoattractants for neutrophils, monocytes, and macrophages, while assembly of the terminal membrane attack complex (C5b-9) inflicts direct cytolytic injury on glomerular resident cells (24, 25). In response, intrinsic mesangial cells and podocytes upregulate production of pro-inflammatory mediators including the monocyte chemoattractant MCP-1/CCL2 and the pleiotropic cytokine IL-6, establishing a positive feedback loop that amplifies leukocyte infiltration (26, 27). Recruited neutrophils undergo NETosis, releasing extracellular traps containing histones and chromatin that cause mechanical and enzymatic damage to the glomerular basement membrane while simultaneously providing nucleic acid ligands for innate pattern recognition receptors, thereby sustaining a self-perpetuating inflammatory circuit (28).

It is important to emphasize that C3, not C5, is the central convergence point of all three complement activation pathways (classical, lectin, and alternative). Cleavage of C3 by C3 convertase generates the anaphylatoxin C3a and the opsonin C3b; subsequent assembly of C5 convertase from C3b leads to C5 cleavage, producing C5a (the most potent anaphylatoxin) and C5b, which initiates formation of the membrane attack complex (C5b-9). In lupus nephritis, the classical pathway is the primary driver of complement activation through C1q binding to immune complexes, but the alternative pathway amplification loop, in which C3b deposits generate additional C3 convertase, substantially amplifies tissue injury. The lectin pathway, activated by pattern recognition molecules binding to altered glomerular glycocalyx, also contributes in a subset of patients. Complement consumption (low serum C3 and C4) remains a standard clinical biomarker in LN, though it reflects systemic rather than intrarenal complement activation and correlates imperfectly with renal disease activity. Emerging complement-targeted therapies, including avacopan (C5a receptor antagonist) and iptacopan (Factor B inhibitor, targeting alternative pathway amplification), are discussed in detail in Section 7.5 (24, 25, 29, 30).

### 2.3 The interferon transcriptional signature: a core pathogenic axis in LN

The interferon gene signature—defined by coordinated upregulation of IFN-stimulated genes (ISGs)—is detectable in 50–75% of patients with SLE and is particularly enriched in those with active renal disease (31, 32). This transcriptional program is principally sustained by IFN-I secreted from pDCs, with additional contributions from IFN-γ produced by T cells and natural killer cells, and from IFN-λ (33). IFN-I exerts pleiotropic immunomodulatory effects including promotion of pDC viability, reduction of B cell activation thresholds, enhancement of antigen-presenting cell function, and suppression of regulatory T cell (Treg) homeostasis (34). The specific intracellular signaling mechanisms and downstream effector programs of both type I and type II interferons operating within the LN kidney are examined in detail in Section 4.1.

Adaptive immune dysregulation in LN involves coordinated disruption of multiple lymphocyte populations whose individual contributions are detailed in Section 3. The CD4+ T cell compartment is skewed toward pro-inflammatory Th1 and Th17 subsets at the expense of Tregs (35), while T follicular helper (Tfh) cells support pathogenic autoantibody production through germinal center reactions (36, 37). CD8+ T cells and double-negative (DN) T cells contribute to tubulointerstitial injury through cytotoxic effector functions (38).

B cell homeostasis is profoundly altered, with plasmablast expansion and elevated BAFF levels accompanying disease flares (39), and intrarenal B cell aggregates contributing to local immune complex formation (40). Disease-associated B cell subsets, including age-associated B cells (ABCs), have been identified through single-cell transcriptomics (41) and are discussed further in Section 3.3.

### 2.4 Patterns of glomerular injury and associated molecular features

The glomerulus is the primary site of immune complex deposition and the principal target of inflammatory injury in lupus nephritis. The pattern, location, and extent of immune complex deposits within the glomerulus largely determine the histological class and clinical presentation. Mesangial deposits (Class II) cause mesangial proliferation with mild proteinuria and hematuria. Subendothelial deposits (Class III/IV) trigger endocapillary hypercellularity, leukocyte infiltration, and activation of the classical complement pathway, producing the full-house immunofluorescence pattern (IgG, IgA, IgM, C3, C1q) characteristic of proliferative LN. Severe proliferative disease may progress to cellular crescent formation, reflecting rupture of the glomerular basement membrane and proliferation of parietal epithelial cells in Bowman’s space. Subepithelial deposits (Class V, membranous LN) activate complement through the membrane attack complex (C5b-9) pathway, injuring podocytes and producing nephrotic-range proteinuria without prominent inflammatory cell infiltration (10, 11, 42, 43).

Podocyte damage constitutes a pivotal determinant of proteinuria magnitude and long-term glomerular prognosis across all histological classes of LN. The combined insults of immune complex-triggered complement activation, oxidative stress, and direct cytokine-mediated injury from TNF-α, IL-6, and IFN-γ drive podocyte foot process retraction, actin cytoskeletal disorganization, and, in advanced cases, physical detachment of podocytes from the underlying glomerular basement membrane. When podocyte loss exceeds a critical density threshold, segmental glomerulosclerosis develops—an irreversible structural lesion that contributes to progressive deterioration of filtration capacity (44, 45).

#### 2.4.1 Lupus podocytopathy

Lupus podocytopathy constitutes a clinically consequential yet frequently underappreciated condition in which patients with SLE manifest nephrotic-range proteinuria and widespread podocyte foot process effacement on ultrastructural examination, without substantial immune complex deposition or proliferative glomerular changes. The histological appearance is indistinguishable from minimal change disease or focal segmental glomerulosclerosis arising in the setting of SLE. Differentiating this entity from Class V (membranous) LN carries direct therapeutic importance, as lupus podocytopathy characteristically demonstrates favorable responses to glucocorticoids and calcineurin inhibitors, in contrast to membranous LN which may necessitate more aggressive immunosuppressive intervention (46, 47).

Proposed pathogenic mechanisms for lupus podocytopathy include circulating permeability factors derived from T cells (possibly analogous to those implicated in idiopathic minimal change disease), elevated levels of soluble urokinase-type plasminogen activator receptor (suPAR), and direct cytokine-mediated podocyte injury (particularly IL-13 and TNF-α). Recent spatial transcriptomic and single-cell studies have begun to identify podocyte stress transcriptional signatures (downregulation of NPHS2/podocin, WT1, SYNPO; upregulation of VEGFA and injury-associated genes) that may distinguish lupus podocytopathy from immune complex-mediated podocyte injury, although dedicated studies remain limited. Within the convergent-to-divergent framework, lupus podocytopathy represents a distinct divergent pathway with specific therapeutic implications (48, 49).

#### 2.4.2 Tubulointerstitial injury

Tubular pathology in LN encompasses a spectrum including epithelial cell vacuolization, brush border loss with cellular flattening, and overt tubular necrosis, resulting from both hemodynamic ischemia secondary to glomerular inflammation and direct immune-mediated tubular cell injury. Interstitial inflammation—marked by infiltration of T lymphocytes, macrophages, and dendritic cells into the tubulointerstitial compartment—is a defining feature of active LN and independently predicts long-term renal functional outcomes. The progressive accumulation of interstitial fibrosis and tubular atrophy (IFTA) reflects irreversible structural remodeling and is quantified by the chronicity index within the ISN/RPS classification framework (48, 50, 51).

### 2.5 The intrarenal immune cell milieu

Macrophages represent the numerically dominant immune cell population within the LN kidney, exhibiting a broad functional continuum extending from classically activated pro-inflammatory states to alternatively activated pro-fibrotic phenotypes—although this binary categorization is now widely recognized as overly reductive, with single-cell profiling data revealing a complex spectrum of intermediate and transitional activation states (see Section 3.4). During active LN, bone marrow-derived monocyte-macrophages accumulate throughout the glomerular and interstitial compartments, elaborating TNF-α, IL-6, and reactive oxygen species (52). As disease evolves toward chronicity, macrophage populations expressing TGF-β1 and pro-fibrotic gene programs become increasingly dominant, promoting progressive tubulointerstitial scarring.

Additional innate immune populations identified within LN kidneys include intrarenal dendritic cells, mast cells, natural killer cells, and innate lymphoid cells (ILCs), with type 2 conventional dendritic cell populations notably enriched in LN biopsy specimens and pDCs sustaining local IFN-I production within the renal parenchyma. These diverse innate immune subsets engage in reciprocal interactions with resident renal epithelial, mesangial, and stromal cells, collectively shaping the inflammatory microenvironment and determining the balance between tissue injury and reparative processes.

A fundamental paradox emerges from the pathogenesis literature summarized above. The conventional linear model of LN—progressing from tolerance breakdown through autoantibody formation, immune complex deposition, complement engagement, and inflammatory cell recruitment—implies a unitary pathogenic trajectory that should logically respond to uniform therapeutic interventions. Yet clinical experience reveals a starkly different picture: patients sharing histologically identical Class IV disease demonstrate dramatically different treatment responses and long-term renal outcomes (12). Emerging single-cell molecular data increasingly indicate that this clinical variability reflects authentic molecular heterogeneity rather than stochastic biological noise. The same ISN/RPS histological class may encompass fundamentally divergent immune infiltrate profiles—with one patient’s Class IV biopsy dominated by IFN-driven macrophage-TEC inflammatory circuits while another’s is characterized by Tfh-B cell networks maintaining ectopic germinal center activity (8, 36). This finding directly challenges the assumption that ISN/RPS classification defines a molecularly homogeneous disease entity, and indicates that morphological assessment, while essential for documenting structural injury patterns, is insufficient as the exclusive foundation for therapeutic decision-making. We contend that a clinically actionable disease classification system for LN must incorporate histological pattern, molecular subtype (defined by the dominant immune pathway activation program), and temporal disease phase (early inflammatory versus established fibrotic), and that the profiling technologies examined in Section 5 are rendering such integrated classification technically achievable, albeit not yet prospectively validated in clinical practice.

The tubulointerstitial compartment warrants special emphasis as an autonomous determinant of renal prognosis in LN. The degree of tubulointerstitial inflammation, tubular atrophy, and interstitial fibrosis predicts long-term renal functional decline more reliably than glomerular activity scores considered in isolation (42). Identification of molecular signatures capturing tubulointerstitial damage—through spatial gene expression profiling and urinary analyte measurement—represents a high-priority objective in contemporary LN biomarker research (53).

## 3 Immune cell populations mediating renal tissue injury in lupus nephritis

The inflammatory milieu within the LN kidney arises from the coordinated yet dysregulated activation of multiple immune cell lineages. Recent advances in single-cell and spatially resolved transcriptomic profiling have fundamentally reshaped understanding of this cellular ecosystem, revealing disease-associated immune cell subsets and complex intercellular communication networks that cannot be discerned through conventional bulk tissue analysis approaches (8).

### 3.1 T helper cell subsets and their pathogenic roles

The CD4+ T helper cell compartment is functionally skewed in LN, featuring disproportionate expansion of Th1 and Th17 effector populations relative to immunosuppressive Tregs (54). Circulating IL-17A concentrations show positive correlation with anti-dsDNA antibody titers and composite SLEDAI disease activity scores during active nephritis episodes (55, 56), and genetic ablation of the IL-23 receptor significantly attenuates nephritis severity in experimental murine models (57). Single-cell transcriptomic profiling has mapped IL-17-producing CD4+ T cells co-expressing the chemokine receptor CXCR3 to areas of active tubulointerstitial inflammation within LN biopsies (8, 48, 55), although it should be noted that context-dependent anti-inflammatory functions of IL-17 have also been documented (58).

T follicular helper (Tfh) cells, defined by CXCR5+PD-1+ICOS+Bcl-6+ surface marker expression, are essential facilitators of germinal center B cell responses and are numerically expanded in SLE (59). Within the LN kidney, Tfh-like cells expressing the chemokine CXCL13 preferentially localize to organized TLS aggregates, and their density correlates with the extent of interstitial damage and poorer clinical outcomes (36). Peripheral T helper (Tph) cells, originally characterized in the inflamed synovium of rheumatoid arthritis (60), may also participate in intrarenal autoimmune responses in LN, though supporting evidence for this role remains preliminary. In the opposing direction, both the numerical abundance and functional suppressive capacity of Tregs are diminished in active SLE, with IFN-I directly suppressing Foxp3 transcription and IL-6 driving phenotypic conversion of Tregs toward a Th17-like effector state (61). Clinical trials of low-dose recombinant IL-2 have demonstrated selective Treg compartment expansion accompanied by clinical improvement in SLE (62).

### 3.2 Cytotoxic T lymphocytes and double-negative T cells

CD8+ cytotoxic T lymphocytes participate in tubulointerstitial tissue destruction, with their infiltration density demonstrating positive correlation with markers of tubular injury and rate of renal function deterioration (63). Single-cell profiling studies have resolved clonally expanded effector CD8+ T cell clusters expressing granzyme B (GZMB) and perforin (PRF1), with oligoclonal T cell receptor repertoire expansions consistent with local antigen-driven clonal selection within LN kidney biopsies (8). Double-negative T cells (CD4−CD8−), characteristically expanded in SLE patients, infiltrate the renal parenchyma and produce IL-17, contributing to amplification of local inflammatory cascades (64).

### 3.3 B lymphocytes and antibody-secreting cells

B cell homeostasis is fundamentally perturbed in SLE, with substantially increased plasmablast and plasma cell frequencies coinciding with disease flares. Single-cell molecular profiling has uncovered an expanded population of double-negative 2 (DN2) B cells—defined by the IgD−CD27−CXCR5−CD21−T-bet+FcRL5+ phenotype—that are selectively enriched in active LN and exhibit heightened propensity for differentiation into autoantibody-secreting plasmablasts (65). Within the LN kidney, B lymphocytes and plasma cells accumulate within the interstitial compartment, occasionally organizing into structured tertiary lymphoid structures (TLS; also referred to as ectopic lymphoid structures, ELS) containing T cells and follicular dendritic cell networks (40). Circulating and tissue levels of B cell activating factor (BAFF) and APRIL are elevated in LN, driven in part by IFN-I-mediated transcriptional upregulation (66). Belimumab, a monoclonal antibody directed against soluble BAFF, reduces circulating plasmablast counts and has demonstrated clinical efficacy in active LN (67).

### 3.4 Mononuclear phagocyte system: monocytes, macrophages, and dendritic cells

Monocytes and macrophages rank among the most numerically abundant immune cells within the LN kidney. Molecular profiling by the AMP consortium has resolved at least three distinct monocyte/macrophage transcriptional clusters: a classically activated inflammatory population defined by FCGR3A, S100A8/A9, and IL1B expression; a tissue-resident homeostatic population; and a phenotypically intermediate population (8). The inflammatory cluster demonstrates selective numerical expansion during active LN. A TREM2+LGMN+ macrophage subset, identified in kidneys with established fibrosis, may play a functional role in mediating the transition from active inflammatory injury to chronic scarring. In other disease contexts, multiomics analysis-assisted deep learning models have demonstrated the capacity to identify macrophage-oriented functional modules as potential therapeutic targets—as exemplified by the discovery of a macrophage-centered therapeutic module in colorectal cancer through integrated multiomics profiling (68)—underscoring the translational potential of computational approaches for dissecting macrophage heterogeneity in inflammatory diseases including LN. Urinary MCP-1/CCL2 stands among the most extensively validated non-invasive molecular indicators of LN activity, demonstrating consistent correlation with histological activity indices and capacity to predict impending disease flares (69, 70). Type 2 conventional dendritic cell populations enriched in LN kidney tissue express elevated levels of HLA-DR and produce IL-12/IL-23, thereby promoting Th1 and Th17 effector responses (8, 71).

### 3.5 Neutrophils, natural killer cells, and innate lymphoid cells

Low-density granulocytes (LDGs), a pathological neutrophil population numerically expanded during active SLE, demonstrate markedly enhanced capacity for NET formation. NETs released from LDGs contain oxidized mitochondrial DNA that serves as a potent stimulus for IFN-I production, establishing a self-reinforcing positive feedback circuit (28, 72). Single-cell transcriptomic analysis has identified LDG-specific transcriptional clusters exhibiting coordinated upregulation of IFN-stimulated genes and NET-associated effector molecules (73). Natural killer cells and innate lymphoid cells constitute numerically minor but potentially functionally significant components of the intrarenal immune infiltrate, although their detailed characterization within the LN kidney remains at an early investigative stage.

The role of neutrophils and NETs in LN pathogenesis extends well beyond IFN-I induction. NETs released in the glomerular microenvironment contain cytotoxic histones (particularly H3 and H4) that directly damage the glomerular basement membrane, endothelial cells, and podocytes. The antimicrobial peptide LL-37 (cathelicidin), a major NET component, complexes with self-DNA to form particles that activate plasmacytoid dendritic cells through TLR9 engagement, further amplifying the type I IFN response. HMGB1, released from NET chromatin, signals through RAGE and TLR4 on resident renal cells, promoting chemokine secretion and leukocyte recruitment. Importantly, NETs also serve as potent activators of the complement system: NET-bound histones activate the alternative complement pathway, while NET-associated properdin stabilizes C3 convertase on the NET scaffold, generating C3a and C5a anaphylatoxins locally. This NET-complement crosstalk creates a feed-forward inflammatory loop that may be particularly relevant in patients with prominent intrarenal neutrophil infiltration (28, 72, 74).

From a multi-omic perspective, single-cell transcriptomic analysis has revealed that LDGs in active SLE express a distinct transcriptional profile enriched for IFN-stimulated genes (ISGs), granule proteins (MPO, ELANE, PRTN3), and NET-associated chromatin remodeling genes (PADI4, HMGB1) (73). These LDG clusters are transcriptionally distinct from mature neutrophils and monocytes, supporting their classification as a pathogenic myeloid population unique to active autoimmune disease. Within the convergent-to-divergent framework, the neutrophil/NET pathway represents a potentially distinct divergent branch that amplifies both the IFN-driven and complement-driven arms of the disease and may identify patients requiring complement-targeted or NET-targeted therapeutic approaches (72, 73, 75).

Several unresolved questions emerge from this cellular analysis. First, whether single-cell-defined subsets represent stable cell states with distinct pathogenic roles or transient activation states along a continuous trajectory remains uncertain, a distinction with direct therapeutic implications. Second, key findings from mouse models (e.g., Th17 pathogenicity, Treg protection) have not been consistently recapitulated in human single-cell studies, possibly reflecting species differences or methodological limitations of human tissue sampling (54, 57, 58). Third, most studies report single time-point data; the temporal dynamics of immune cell infiltration, which cells arrive first, persist through treatment, and predict relapse, remain largely unknown and require longitudinal profiling to elucidate.

### 3.6 Cellular landscape summary

In summary, single-cell and spatial profiling have identified key pathogenic themes in LN: expansion of inflammatory monocyte-derived macrophages and IFN-activated TECs; the role of Tfh cells and DN2 B cells in autoantibody responses; and LDG-mediated NETosis amplifying IFN signaling. These insights provide the cellular foundation for the biomarker and therapeutic discoveries discussed in subsequent sections.

## 4 Cytokine signaling networks and intracellular pathway architecture in lupus nephritis

Cytokine signaling networks function as the molecular communication system through which immune cells orchestrate inflammatory processes within the LN kidney. A thorough understanding of the cytokine milieu and intracellular signal transduction cascades is prerequisite for the proper interpretation of single-cell profiling data and the rational identification of druggable therapeutic targets. This section presents a focused overview of the principal cytokine families and signaling nodes with demonstrated roles in LN pathobiology.

### 4.1 Type I and type II interferon signaling pathways

IFN-I (predominantly IFN-α subtypes) engages the heterodimeric IFNAR1/IFNAR2 receptor complex, triggering phosphorylation of JAK1 and TYK2 kinases and subsequent assembly of the ISGF3 transcription factor complex (comprising STAT1, STAT2, and IRF9), which translocates to the nucleus to initiate transcription of ISGs including OAS1, MX1, IFIT1, and CXCL10 (76). Within LN kidney biopsies, active IFN-I signaling is demonstrable not only in infiltrating immune cell populations but also in resident tubular epithelial cells, podocytes, and mesangial cells (48). IFN-γ (type II IFN), produced predominantly by Th1 cells, cytotoxic CD8+ T lymphocytes, and natural killer cells, signals through the JAK1/JAK2 pathway to generate STAT1 homodimer complexes that induce macrophage classical activation and drive expression of CXCR3-binding chemokines CXCL9, CXCL10, and CXCL11 (77). Urinary CXCL10 has emerged as one of the most sensitive non-invasive indicators of intrarenal IFN-γ pathway activity, demonstrating correlation with both histological classification and treatment response (53). The convergent engagement of STAT1 by both IFN-I and IFN-γ signaling pathways establishes a synergistic pro-inflammatory transcriptional program within the LN kidney.

### 4.2 TNF-α, IL-6, and JAK-STAT signal transduction

TNF-α produces context-dependent biological effects in LN: within the renal microenvironment, it signals predominantly through TNFR1 to activate canonical NF-κB pathways and drive transcription of adhesion molecules, chemokines, and pro-apoptotic effectors (78). Notably, NF-κB activation in LN extends well beyond TNF-α stimulation; immune complex engagement, Toll-like receptor ligands, and complement-derived C5a all independently feed into canonical and non-canonical NF-κB signaling cascades. IL-6, transducing signals through either membrane-anchored or soluble IL-6 receptor in partnership with the gp130 co-receptor and activating the JAK1/JAK2-STAT3 axis, promotes Th17 lineage commitment, drives B cell terminal differentiation into antibody-secreting cells, and stimulates hepatic acute phase protein production (79). Single-cell transcriptomic analysis has identified a STAT3-activated macrophage population in LN kidneys characterized by expression of IL6, CSF1, and VEGFA. JAK pathway inhibitors—particularly baricitinib and upadacitinib, which attenuate signaling downstream of IFNAR, IL-6R, IL-2R, and additional cytokine receptors—are currently under active clinical evaluation in LN (80) (see Section 7.4).

### 4.3 Inflammasome activation, TGF-β signaling, and B cell survival pathways

#### 4.2.1 Pathway redundancy and crosstalk

The cytokine and signaling pathway landscape reviewed above exposes a core challenge for mechanism-directed therapy in LN: pervasive pathway redundancy and extensive inter-pathway crosstalk. IFN-I, IFN-γ, IL-6, TNF-α, and IL-17 do not function as discrete linear signaling cascades; instead, they constitute a densely interconnected network featuring multiple positive feedback circuits—IFN-I transcriptionally upregulates BAFF, thereby sustaining B cell survival and autoantibody production. Simultaneously, IL-6 drives Th17 lineage commitment, generating IL-17 that further amplifies neutrophil recruitment to the kidney. In turn, NETosis by infiltrating neutrophils liberates mitochondrial DNA that re-engages IFN-I production through innate pattern recognition receptors (28, 34, 66). This network topology carries two important implications that remain insufficiently addressed in current drug development paradigms. First, pharmacological blockade of any single network node may prove inadequate when redundant parallel pathways compensate—a plausible explanation for the modest effect sizes observed in clinical trials of single-target biologics in LN. Second, the dominant signaling node may vary between individual patients and even fluctuate within the same patient over time, implying that sequential or combinatorial mechanism-directed therapy guided by real-time molecular monitoring may ultimately be required for durable disease control. The molecular disease subtyping framework discussed in Sections 5 and 7 represents a potential approach to managing this complexity, though its clinical realization depends on rapid-turnaround biomarker assays not yet available for routine deployment.

NLRP3 inflammasome assembly within renal macrophages and mesangial cells drives caspase-1-mediated proteolytic maturation of IL-1β and IL-18, which in turn promote Th17 lineage commitment and amplify IFN-γ elaboration (81). TGF-β1, the master regulatory cytokine driving renal fibrogenesis, signals through ALK5/TβRII receptor complexes to activate SMAD2/3 transcriptional programs that induce collagen and fibronectin matrix deposition, epithelial-to-mesenchymal transition, and myofibroblast differentiation (82). In single-cell transcriptomic datasets, TGF-β pathway target gene signatures demonstrate selective enrichment within the TREM2+ pro-fibrotic macrophage population. BAFF and APRIL, found at elevated concentrations in both serum and renal tissue of LN patients, sustain B cell and long-lived plasma cell viability through NF-κB2 signaling; dual BAFF/APRIL blockade using the TACI-Fc fusion protein telitacicept has yielded encouraging results in a phase 2b randomized trial in active SLE (83).

Before reviewing individual platforms and findings, it is important to establish a critical framework for interpreting the rapidly expanding single-cell and spatial omics literature in LN. We propose that published studies be evaluated along four dimensions that collectively determine their translational potential: (i) cohort representativeness: whether the study population reflects the demographic and clinical diversity of LN patients globally, or is drawn from a narrow demographic subgroup at a single center; (ii) analytical reproducibility, whether key findings (cell type clusters, gene signatures, communication networks) have been independently replicated in separate cohorts with different technical pipelines; (iii) clinical correlation, whether molecular findings are linked to clinically meaningful endpoints (treatment response, renal survival, flare prediction) rather than solely to histological descriptors; and (iv) actionability, whether the findings suggest a specific clinical decision that would differ from current standard of care. Applying this framework to the studies reviewed below reveals that most current work achieves strong performance on dimensions (i) through (iii) but remains at an early stage on dimension (iv), a gap that should guide the design of future studies.

### 4.4 mTOR pathway and immunometabolic reprogramming

The mTOR signaling pathway exhibits dysregulated activation in SLE immune cells, with constitutive mTORC1 activity in T lymphocytes promoting a pro-inflammatory functional phenotype. mTORC1 activation preferentially supports Th17 lineage commitment while simultaneously suppressing Treg development; pharmacological mTOR inhibition with sirolimus restores T cell subset balance and reduces circulating anti-dsDNA antibody titers (84). Immunometabolic reprogramming—the shift from mitochondrial oxidative phosphorylation to aerobic glycolysis within immune cells resident in the inflamed kidney—is now appreciated as both a consequence and an active perpetuator of chronic inflammation (85, 86). Recent mechanistic studies have established that mTORC1-mediated glycolytic reprogramming in CD4+ T cells drives Th17 polarization while impairing Treg suppressive function during active SLE (85, 86). In parallel, macrophage populations within the inflamed LN kidney undergo metabolic rewiring toward aerobic glycolysis, augmenting pro-inflammatory cytokine output at the expense of efferocytosis and tissue repair capacity (87). Pharmacological targeting of metabolic control points—including 2-deoxy-D-glucose (glycolysis inhibitor) and metformin (AMPK pathway activator)—has demonstrated therapeutic efficacy in murine lupus models, establishing immunometabolic reprogramming as a pharmacologically tractable axis in LN (85–87).

## 5 High-resolution molecular profiling technologies in lupus nephritis: mapping heterogeneity and advancing disease stratification

The deployment of single-cell and spatially resolved omics technologies to investigate LN marks a qualitative transformation in molecular disease characterization. These methodologies permit simultaneous transcriptomic profiling of thousands to millions of individual cells, resolution of rare and transitional cellular states, quantitative inference of intercellular communication networks, and—in the case of spatial transcriptomic platforms—retention of the tissue architectural context that is sacrificed during conventional dissociation-based single-cell preparation. This section surveys the principal technological platforms and their major contributions to LN biology, molecular biomarker discovery, and the developing framework for molecular disease stratification.

**Table 2** provides a comprehensive summary of the major multi-omic platforms applied to LN research, including cohort sizes, principal findings at the cellular, protein, and molecular levels, biomarker candidates identified, clinical correlations, and associated references. This tabular overview is designed to complement the detailed narrative discussion of each platform in Sections 5.1–5.3, allowing readers to rapidly compare the contributions and limitations of different multi-omic approaches (8, 88). Key novel discoveries from these multi-omic studies are summarized in **Figure 2**.

**Table 2:** Multi-omic approaches in lupus nephritis: platforms, key findings, and references.

Technology/platform	Key study	Cohort	Principal findings	Biomarker candidates	Clinical correlations	Limitations	Ref
scRNA-seq (10x Chromium)	Arazi et al., 2019 (AMP Phase 1)	24 LN biopsies + matched PB	Identified 21 cell clusters; CD16+ inflammatory macrophages expanded in proliferative LN; IFN-stimulated TECs correlating with activity; fibroblast activation in Class IV	IFN-TEC score; macrophage cluster abundance	IFN-TEC correlates with histological activity; fibroblast signature predicts tubular atrophy	Dissociation bias; limited cohort diversity; cross-sectional	(8)
scRNA-seq	Der et al., 2019	LN kidney + skin biopsies	IFN and fibrosis transcriptional programs in TECs; cross-tissue IFN signature in skin and kidney	Skin IFN score as renal activity surrogate	IFN-TEC program correlates with renal activity	Small sample size; limited to IFN pathway	(47)
CITE-seq (multi-modal)	AMP Phase 2 (ongoing)	Expanded LN cohort with paired ATAC-seq, CITE-seq, VDJ	Surface protein-RNA integration; IRF1/IRF7 TF motifs in pDCs; BATF/NFAT in T cells; clonal TCR expansions	TF activity scores; clonotype-based monitoring	TF programs linked to histological class and outcomes	Complex data integration; ongoing validation	(8, 74)
Spatial transcriptomics (Visium)	Multiple groups 2022-2025	10–30 LN biopsies per study	Tubulo-immune niche (IFN-TEC + macrophage co-localization); TLS mapped; glomerular vs TI compartmentalization	Spatial niche scores	TI niche correlates with injury score independently of class	50 um resolution (multi-cell); FFPE optimization ongoing	(89, 90)
scATAC-seq	AMP Phase 2	Paired with scRNA-seq from LN biopsies	Open chromatin at IRF1/IRF7 in pDCs/macrophages; AP-1/KLF in fibroblasts; BATF/NFAT in T cells	TF accessibility signatures	Chromatin states predict cell activation programs	Low per-cell coverage; requires integration with RNA	(8, 74)
Urinary proteomics	Fava et al., 2020/2022	30 LN patients with matched biopsies	Urinary CXCL10 reflects intrarenal IFN-gamma activity; IL-16 correlates with lymphocyte infiltration; sCD163 with macrophage activation	CXCL10, sCD163, IL-16, ALCAM	CXCL10 correlates with histological activity and treatment response	Hydration variability; no consensus on processing protocols	(52, 104)
Metabolomics	Multiple groups 2021-2025	Variable (30–200 patients)	Altered energy metabolism, nucleotide turnover, oxidative stress in active LN; glycolytic shift in immune cells	Urinary metabolite panels	Metabolic profiles distinguish active from inactive LN	Confounders (diet, drugs); limited standardization	(84, 85, 94)
Epigenomics (DNA methylation)	Multiple groups 2019-2024	SLE CD4+ T cells, LN biopsies	Hypomethylation of IFN loci (IFIT1, IFI44L, MX1) and co-stimulatory genes (CD70, CD11a) in SLE T cells	IFN methylation score	Methylation signatures stable across disease states; may underlie IFN chronicity	Bulk tissue; cell-type deconvolution needed	(95, 96)
cfDNA fragmentomics	Chan et al., 2014	Active SLE vs controls	Aberrant fragment-size profiles, hypomethylation, altered genomic representations in cfDNA from active SLE	cfDNA fragment profiles; mt-cfDNA levels	cfDNA alterations correlate with disease activity	Early-stage; no LN-specific validation yet	(105, 106)

### 5.1 Single-cell transcriptomics: technological platforms and LN applications

Single-cell RNA sequencing (scRNA-seq) resolves the transcriptomes of individual cells using one of three principal methodological approaches: barcoded droplet encapsulation (10x Genomics Chromium platform), plate-based full-length transcript sequencing (Smart-seq2/3), or combinatorial split-pool ligation strategies (SPLiT-seq, Parse Biosciences). Each technological platform entails characteristic trade-offs among cellular throughput, transcript capture efficiency, and per-gene detection sensitivity that determine the scope of biological questions amenable to investigation. Within the LN research field, droplet-based approaches—particularly the 10x Genomics platform—have achieved the broadest adoption, driven by their capacity to simultaneously profile tens of thousands of cells per sample, rendering them particularly well-suited to comprehensive characterization of the cellular diversity present in kidney biopsy specimens and matched peripheral blood immune compartments.

The landmark AMP RA/SLE Network Phase 1 investigation, published in 2019, constituted the first systematic large-scale deployment of scRNA-seq technology to LN kidney biopsy tissue (8). Profiling of 24 biopsy specimens across ISN/RPS Classes II through V resolved 21 transcriptionally distinct cell clusters comprising both infiltrating immune and resident non-immune cell populations. Among the principal findings were: resolution of an inflammatory CD16+ monocyte-derived macrophage cluster selectively expanded in proliferative LN classes; identification of an IFN-stimulated tubular epithelial cell (IFN-TEC) transcriptional program whose abundance correlated with histological activity scores; delineation of a non-classical NK/ILC population enriched in membranous LN biopsies; and recognition of an activated fibroblast transcriptional signature in Class IV specimens predictive of subsequent tubular atrophy. The expanded AMP Phase 2 initiative has incorporated paired single-cell chromatin accessibility profiling (scATAC-seq), combined surface protein-transcriptome measurement (CITE-seq), and T cell/B cell receptor repertoire sequencing into a broadened longitudinal biopsy cohort, with findings progressively entering the peer-reviewed literature (8, 53, 88).

Independent scRNA-seq investigations of LN kidneys have corroborated and expanded upon the original AMP observations. Der et al. deployed single-cell transcriptomic profiling across matched LN kidney biopsies and skin tissue specimens, identifying type I IFN and fibrosis-associated transcriptional programs in both tubular epithelial cells and keratinocytes that demonstrate coordinate upregulation during active disease (48). Complementing these single-cell investigations, Bulusu et al. conducted bulk transcriptomic profiling of renal tissue and peripheral blood in a prospective cohort of proliferative LN patients, identifying molecular signatures that distinguished treatment responders from non-responders and predicted therapeutic outcomes (89). Integration of T cell receptor repertoire sequencing with scRNA-seq data has additionally revealed clonally expanded T cell populations preferentially enriched in kidney tissue relative to matched blood, suggesting antigen-driven local clonal selection (8). Taken together, these investigations have constructed a cellular taxonomy of LN that exceeds the molecular resolution achievable through histological classification and is beginning to influence selection of therapeutic targets and disease monitoring strategies.

### 5.2 CITE-seq and multi-modal single-cell molecular profiling

Cellular indexing of transcriptomes and epitopes by sequencing (CITE-seq) permits concurrent measurement of both the full transcriptome and surface protein expression of individual cells through antibody-oligonucleotide conjugate labeling (90). This multimodal technology effectively bridges conventional flow cytometry-based immunophenotyping with unbiased transcriptomic characterization, enabling higher-confidence cell type assignment and detection of protein-RNA discordances—instances where messenger RNA abundance fails to predict surface protein levels, a phenomenon frequently observed for immune cell activation markers and cytokine receptors. In the context of LN, CITE-seq profiling of peripheral blood mononuclear cells has identified surface protein-defined immune subsets harboring distinctive transcriptional programs, notably including a CXCR5hiPD-1hiICOShi Tfh population whose transcriptomic features—including BCL6 and CXCL13 expression—predict renal disease activity and circulating anti-dsDNA antibody concentrations.

Additional multi-modal single-cell technologies encompass simultaneous transcriptome and chromatin accessibility measurement (10x Multiome), paired RNA and TCR/BCR repertoire sequencing (10x VDJ), and REAP-seq for concurrent RNA and proteomic profiling. Integration of these complementary modalities within the AMP Phase 2 analytical framework has permitted investigators to establish direct links between gene regulatory programs—characterized by open chromatin at transcription factor binding sites—and cell type-specific gene expression patterns in LN, identifying candidate master transcription factor regulators of pathogenic cell states, including IRF1 and BATF in inflammatory macrophage populations and TBX21 in effector T cell subsets. These regulatory network findings provide mechanistic rationale for therapeutic strategies directed at transcription factor activity or the upstream signaling pathways that control it.

### 5.3 Spatial transcriptomics: maintaining tissue architectural context

Spatial transcriptomic platforms measure gene expression while simultaneously recording the two-dimensional tissue coordinates of each measurement site, thereby enabling direct correlation of transcriptional programs with histological structures and inflammatory microenvironments. Two principal technological categories exist: array-based capture methods that quantify transcripts at predefined spots on a glass-mounted capture array (10x Visium, generating ~50 µm spots each encompassing approximately 10–20 cells), and subcellular-resolution single-molecule imaging methods that detect individual transcript molecules *in situ* (MERFISH, seqFISH+, Xenium, CosMx SMI)—the latter achieving authentic single-cell spatial resolution with capacity to simultaneously detect hundreds of distinct transcript species (91, 92).

Deployment of array-based spatial transcriptomics (10x Visium) on LN kidney biopsy sections has begun to systematically map the spatial organization of immune infiltrates in relation to nephron compartments and histological lesion types, informed by reference single-cell atlases of the healthy human kidney that define the normal cellular and epigenomic landscape (93). Computational integration of spatial expression data with matched single-cell RNA-seq profiles has demonstrated that inflammatory cell populations—including monocyte-derived macrophages and activated T lymphocytes—spatially co-localize with regions of active glomerulitis and tubulitis, whereas fibroblast activation transcriptional programs show spatial enrichment in areas of established tubular atrophy and interstitial fibrosis. Spatial proximity analysis has additionally defined a tubulo-immune niche, characterized by intimate physical apposition of IFN-stimulated TECs and activated macrophages, that correlates with tubulointerstitial injury scores independently of glomerular histological class and may constitute a self-sustaining unit of chronic inflammation with implications for both prognostication and therapeutic intervention.

Single-molecule *in-situ* spatial profiling platforms—including Slide-seq, MERFISH, seqFISH+, and CosMx SMI—provide subcellular measurement resolution that may eventually enable comprehensive mapping of immune cell distributions at the scale of individual glomeruli and tubular segments (94). Although systematic application of these ultra-high-resolution technologies to human LN kidney tissue remains in its early phases, their inherent capacity to resolve direct intercellular contacts within organized inflammatory structures—such as plasma cell niches embedded within TLS aggregates and macrophage–epithelial contact zones—positions them as potentially powerful tools for future spatially resolved immunopathological investigations in LN. Standardization of spatial transcriptomic processing workflows for clinical kidney biopsy material, including technical optimization for formalin-fixed paraffin-embedded (FFPE) tissue specimens, remains an active area of methodological development.

### 5.4 Cross-platform multi-omics integration and molecular disease classification

Integration of data from multiple omics layers, combining, for example, bulk or single-cell transcriptomics with proteomics, metabolomics, and epigenomics, provides a more comprehensive view of disease biology than any single platform and can reveal regulatory mechanisms not apparent from transcriptomics alone. In LN, several multi-omics integration studies have been reported in the past five years, though most remain limited by sample size and cross-sectional design. Banchereau et al. performed transcriptomic and proteomic profiling of peripheral blood from a longitudinal SLE cohort and identified molecular stratification modules, including an IFN-high/neutrophil-high subgroup with disproportionate renal involvement, that predicted flare risk independently of clinical scores (73).

Fava et al. integrated urinary proteomics with single-cell genomic data from matched kidney biopsies in a cohort of 30 LN patients and identified an IFN-γ response gradient in renal tissue that was reflected in the urinary proteome, with urinary CXCL10 as the strongest non-invasive correlate of intrarenal IFN-γ activity (53). This study established a proof of concept for using urine as a “liquid kidney biopsy” to capture molecular events within the inflamed kidney without invasive sampling. Multi-omics profiling of peripheral blood from SLE and LN patients has identified stratification modules, incorporating plasmablast, low-density granulocyte, and inflammatory monocyte programs, that associate with histological LN class and renal disease severity, providing a conceptual basis for blood-based molecular classification to guide monitoring and biopsy decisions (16, 73).

Computational methods for multi-omics integration include weighted gene co-expression network analysis (WGCNA), multi-omics factor analysis (MOFA+), similarity network fusion (SNF), and graph neural network approaches that can learn cross-modal associations in an unsupervised or weakly supervised manner. Application of MOFA+ to a dataset integrating scRNA-seq, ATAC-seq, and surface proteomics from LN peripheral blood identified a latent factor capturing the plasmablast-IFN axis that explained the largest proportion of disease-related variance and was significantly associated with renal flare within six months. These computational approaches are transitioning from proof-of-concept demonstrations to tools that can be prospectively validated in clinical cohorts, though sample sizes required for robust multi-omics integration typically exceed those available from single-center studies.

A candid assessment of the multi-omics integration literature in LN reveals both genuine progress and significant limitations. On the positive side, integration studies have demonstrated that no single omics layer captures the full complexity of LN biology, transcriptomic endotypes defined by RNA expression do not perfectly predict proteomic or metabolomic profiles, implying post-transcriptional regulatory mechanisms that would be invisible to transcriptomics alone (53, 73). On the other hand, multi-omics integration in LN currently suffers from what might be termed the ‘n problem’: the statistical methods available for cross-modal integration (MOFA+, SNF, graph neural networks) require sample sizes substantially larger than those available from any single study to produce stable, generalizable results. Most published multi-omics LN studies have profiled 20–50 patients, sufficient for discovery but insufficient for the robust validation that clinical translation demands. International data-sharing consortia and federated analysis approaches that allow integration across centers without requiring raw data transfer represent a critical infrastructure need for the field.

#### 5.4.1 Pathway-specific tissue injury programs delineated by multi-omic integration

Multi-omic integration has enabled the identification of molecularly distinct intrarenal disease signatures, or divergent tissue programs(molecular endotypes, i.e., molecularly defined disease subtypes), that define subgroups of LN patients with different dominant pathogenic mechanisms. Rather than viewing LN as a single entity, these signatures suggest that the same ISN/RPS histological class can harbor fundamentally different molecular diseases. Below, we organize the principal divergent signatures identified to date, integrating pathophysiology, multi-omic evidence, candidate biomarkers, and potential therapeutic targets for each (8, 16, 88).

##### 5.4.1.1 Intrarenal ectopic germinal center and tertiary lymphoid structure program

Organized TLS composed of B cells, plasma cells, Tfh-like cells, and follicular dendritic cell networks have been documented in a proportion of LN biopsies, with preferential localization to the tubulointerstitial compartment. Single-cell transcriptomic analysis has demonstrated CXCL13-expressing Tfh-like cells and CXCR5+ B cells co-localizing within these organized aggregates, providing molecular evidence for local germinal center-like reactions and *in situ* autoantibody generation (36, 40). Spatial gene expression profiling has confirmed the organized cellular architecture of these formations and established their association with interstitial inflammation severity scores. Urinary CXCL13 concentrations have been proposed as a potential non-invasive indicator of intrarenal TLS activity. From a therapeutic perspective, this tissue injury program suggests potential benefit from B cell-depleting strategies (obinutuzumab, CAR-T cells) or BAFF/APRIL pathway blockade (belimumab, telitacicept) aimed at disrupting the ectopic germinal center reaction.

##### 5.4.1.2 IFN-γ-mediated macrophage–tubular epithelial cell co-activation circuit

The AMP consortium delineated a prominent macrophage-TEC inflammatory intercellular communication network mediated through IFN-γ signaling and CXCL10/CXCR3 chemokine receptor interactions (8, 53). Within this tissue program, IFN-stimulated TECs—characterized by elevated expression of ISGs including MX1, IFIT1, and CXCL10—engage in reciprocal signaling with inflammatory bone marrow-derived macrophages (FCGR3A+, S100A8/A9+, IL1B+), establishing a self-perpetuating inflammatory microenvironment. This co-activation circuit demonstrates particular numerical expansion in proliferative (Class III/IV) LN. Urinary CXCL10 functions as a non-invasive molecular indicator reflecting intrarenal IFN-γ pathway activity and demonstrates correlation with both histological activity scores and treatment responsiveness (53). From a therapeutic standpoint, this tissue program identifies patients potentially most responsive to IFN pathway blockade (anifrolumab) or JAK pathway inhibition (baricitinib, upadacitinib).

##### 5.4.1.3 Neutrophil/NET-driven tissue injury program

A proportion of LN patients demonstrate prominent intrarenal neutrophil accumulation accompanied by active NETosis. Low-density granulocytes (LDGs), characteristically expanded during active SLE, display markedly enhanced NET-generating capacity and release oxidized mitochondrial DNA that engages TLR7/9-mediated innate sensing to stimulate IFN-I production, establishing a self-amplifying positive feedback circuit (28, 72). Single-cell molecular profiling has resolved LDG-specific transcriptional clusters exhibiting coordinated upregulation of ISGs alongside NET-associated effector genes including PADI4, MPO, and ELANE (73). Released NETs additionally engage the complement system—particularly the alternative pathway amplification loop—directly damage podocytes and endothelial cells, and provide a continuous source of nuclear autoantigens. Urinary NET degradation products (MPO-DNA complexes, citrullinated histone H3) are being evaluated as potential molecular indicators. Therapeutically, this tissue injury program may respond to complement pathway inhibition (avacopan), PAD4 enzymatic inhibitors (currently in preclinical development), or DNase-based strategies designed to accelerate NET degradation and clearance.

##### 5.4.1.4 Podocyte-autonomous injury and lupus podocytopathy

Lupus podocytopathy represents a distinct entity in which nephrotic-range proteinuria in SLE results from diffuse podocyte foot process effacement in the absence of significant subepithelial or subendothelial immune complex deposition. This entity is clinically important because it responds to glucocorticoids and calcineurin inhibitors rather than conventional LN immunosuppression. Proposed pathogenic mechanisms include T cell-derived circulating permeability factors, elevated soluble urokinase-type plasminogen activator receptor (suPAR) levels, and direct cytokine-mediated podocyte injury (IL-13, TNF-α). Single-cell studies have identified podocyte stress signatures (NPHS2 downregulation, WT1 loss, VEGFA upregulation) in spatial transcriptomic datasets, though dedicated studies of lupus podocytopathy are limited. Recognition of this pathway is critical for avoiding unnecessary intensification of immunosuppression in patients whose proteinuria is driven by podocyte dysfunction rather than immune complex-mediated glomerulonephritis (46, 47, 49).

##### 5.4.1.5 TREM2+ macrophage-orchestrated fibrotic remodeling program

Progressive tubulointerstitial fibrosis represents the terminal common pathway leading to irreversible loss of renal function in LN. Multi-omic profiling data have resolved a TREM2+LGMN+ pro-fibrotic macrophage population preferentially enriched in biopsies exhibiting high chronicity indices, which elaborates TGF-β1 and drives fibroblast-to-myofibroblast phenotypic conversion with subsequent extracellular matrix deposition (8). Spatial gene expression mapping has localized these pro-fibrotic macrophage populations to regions of established tubulointerstitial scarring. Epithelial-to-mesenchymal transition (EMT) transcriptional programs, identifiable through single-cell pseudotemporal trajectory analysis, further contribute to myofibroblast pool expansion. Urinary indicators of fibrotic activity—including TGF-β1, MMP-7, and collagen III degradation fragments—may function as non-invasive surrogates for ongoing tissue remodeling. From a clinical perspective, identifying patients harboring an established fibrotic transcriptional signature is consequential for prognostic assessment (such patients derive limited benefit from intensification of anti-inflammatory therapy) and may inform development of anti-fibrotic agents (pirfenidone, nintedanib analogues) as adjunctive therapeutic strategies.

Beyond these unsupervised integration methods, supervised machine learning (ML) and deep learning (DL) approaches represent a logical next step for multi-omics analysis in LN, yet their application to this disease remains notably underdeveloped relative to oncology and even other nephropathies. In principle, ML-based feature selection methods such as LASSO regression, elastic net, and gradient-boosted trees could be applied to the urinary proteomic datasets described in Section 5.5 to distill high-dimensional signatures into parsimonious biomarker panels, for instance, identifying minimal analyte combinations from among CXCL10, sCD163, IL-16, and ALCAM that approximate the diagnostic performance of full proteomic profiles for distinguishing active from inactive LN. However, to our knowledge, systematic ML-driven optimization of urinary LN biomarker panels with independent cohort validation has not yet been reported, and claims of ML-optimized panels in this disease remain largely aspirational rather than evidence-based. In computational pathology, convolutional neural networks (CNNs) have shown promise in other nephropathies such as diabetic nephropathy and IgA nephropathy for automated morphological feature extraction, but their systematic application to LN-specific tasks remains at an early proof-of-concept stage with small, single-center cohorts. Several factors specific to LN constrain the near-term utility of ML/DL approaches. The sample sizes typical of LN cohorts, tens to low hundreds of patients, as discussed above, are fundamentally insufficient for training robust deep learning models, and overfitting remains a pervasive risk that transfer learning from larger datasets in other renal diseases only partially mitigates. Most computational analyses of LN multi-omics data have accordingly employed unsupervised or weakly supervised methods (MOFA+, SNF, WGCNA) precisely because labeled training data are too limited for fully supervised approaches. The lack of standardized digital pathology workflows across institutions introduces technical variability that degrades model generalizability, and the interpretability deficit of deep learning models, their “black box” nature, poses a barrier to clinical adoption and regulatory approval. We therefore regard ML/DL-based precision nephrology in LN as an important aspiration that requires, as prerequisites, the large-scale international collaborative datasets and federated learning frameworks discussed above, combined with prospective clinical validation. Until these prerequisites are met, the field should resist the temptation to overstate the current contribution of artificial intelligence to LN biomarker discovery.

### 5.5 Urine-based molecular profiling: toward a non-invasive kidney biopsy

The kidney is unique among solid organs in that it continuously exports cellular and molecular material into the urine, providing a non-invasive window into renal immune events. Urinary sediment contains viable immune and tubular epithelial cells that can be profiled by flow cytometry, transcriptomics, or proteomics without renal biopsy. Single-cell RNA sequencing of urine sediment cells (urinary scRNA-seq) has been applied to LN with promising results: integrated urine proteomics and renal single-cell genomics have demonstrated that urinary immune cell and protein signatures from LN patients recapitulate the transcriptional programs of their intrarenal counterparts, with urinary CXCL10 emerging as the strongest non-invasive correlate of intrarenal IFN-γ activity (53). In parallel, Perez et al. applied multiplexed single-cell RNA sequencing (mux-seq) to peripheral blood mononuclear cells from 162 SLE patients and 96 healthy controls, profiling over 1.2 million cells and identifying cell type-specific genetic associations and disease-associated transcriptional programs in circulating immune cells that complement tissue-based and urinary findings (88).

Beyond viable cells, urine contains extracellular vesicles (EVs), free RNA, proteins, and metabolites released from the inflamed kidney. Urinary EV proteomics has identified candidate biomarkers including podocyte-derived nephrin fragments, proximal tubule-derived NGAL, and macrophage-derived CD63+ vesicles enriched in cytokine cargo. Urinary microRNA profiling has demonstrated consistent dysregulation of miR-21, miR-150, and miR-29c in active LN, with miR-21 and miR-150 upregulated and miR-29c downregulated compared with controls, with miR-150 showing particular promise as a marker of glomerular inflammation owing to its enrichment in immune cell-derived urinary EVs (95). Urinary metabolomics has identified alterations in energy metabolism, nucleotide turnover, and oxidative stress pathways in active LN; however, careful attention to confounding factors and analytical standardisation is essential before urinary metabolic profiles can be applied as reliable clinical biomarkers (96).

A significant practical advantage of urine-based biomarkers is their suitability for serial monitoring, unlike kidney biopsies, which are limited in frequency by procedural risk, urine samples can be collected at each clinic visit, enabling longitudinal tracking of molecular disease activity. Prospective longitudinal biomarker cohort initiatives are systematically collecting serial urine samples with paired clinical and biopsy data to validate candidate urinary biomarker panels in adequately powered and ethnically diverse cohorts. Challenges to clinical implementation include inter-individual variability in urine concentration, need for standardized collection and processing protocols, and the requirement for multiplexed rather than single-analyte measurement to achieve adequate sensitivity and specificity.

It is important, however, to offer a candid assessment of how far urine-based “liquid kidney biopsy” approaches remain from routine clinical application. While the conceptual appeal of non-invasive molecular monitoring is undeniable, several practical barriers have not yet been resolved. First, there is no consensus on specimen handling: collection timing (first morning void versus random spot), preservative use, centrifugation protocols, and storage conditions all affect analyte recovery, and standardized operating procedures accepted across clinical laboratories have not been established (96). Second, urinary biomarker concentrations are influenced by hydration status, urinary tract infections, menstrual contamination, concurrent medications, and renal function itself, introducing confounders that are difficult to control outside of research settings. Third, most published urinary biomarker studies have been conducted in single-center or single-ethnicity cohorts with sample sizes of 30–99 patients; multi-center, multi-ethnic validation, essential for establishing generalizable diagnostic thresholds, remains limited. Fourth, the analytical platforms used for urinary scRNA-seq, EV proteomics, and metabolomics are technically demanding, expensive, and not yet available in routine clinical laboratories; translating these research tools into rapid-turnaround clinical assays requires substantial assay simplification and cost reduction that has not been achieved. At present, urine-based molecular profiling in LN should be regarded as an active and promising area of translational research rather than a near-term clinical reality, and the literature should be interpreted with this distinction clearly in mind.

### 5.6 Epigenomic remodeling and chromatin accessibility profiling in LN

Epigenomic remodeling, alterations in DNA methylation, histone modification, and chromatin accessibility, contributes to the persistent dysregulation of immune cell gene expression in SLE and LN. Genome-wide DNA methylation studies have identified hypomethylation of interferon-regulated loci (IFIT1, IFI44L, MX1) and co-stimulatory molecule genes (CD70, CD11a) in SLE CD4+ T cells, contributing to T cell hyperactivation and IFN pathway amplification (97, 98). These methylation signatures are stable across disease states and may underlie the chronicity of IFN elevation even during apparent clinical remission.

Chromatin accessibility profiling by ATAC-seq (assay for transposase-accessible chromatin with sequencing) at the single-cell level (scATAC-seq) has been integrated with scRNA-seq in LN kidney biopsies in the AMP Phase 2 initiative, enabling inference of active transcription factor networks from open chromatin peaks. IRF1 and IRF7 binding motifs are enriched in open chromatin regions of pDCs and IFN-stimulated macrophages in LN biopsies, consistent with IFN pathway activation; BATF and NFAT motifs are enriched in effector T cell clusters; and AP-1 and KLF motifs characterize activated fibroblast populations in fibrotic biopsies (8, 88). These transcription factor programs are potential druggable nodes that may allow more targeted intervention than broad cytokine blockade.

Histone modification profiling by CUT&RUN or CUT&TAG at the bulk or single-cell level is beginning to be applied to LN kidney biopsy material, though the small tissue quantities available from diagnostic biopsies limit throughput.

### 5.7 Intercellular communication network inference and ligand-receptor interaction analysis

Single-cell transcriptomic data can be leveraged to infer cell-cell communication through ligand-receptor interaction analysis (ligand-receptor interactome, i.e., the network of cell-to-cell communication signals), using tools such as CellChat, NicheNet, CellPhoneDB, and LIANA. These approaches identify statistically enriched ligand-receptor pairs between cell type clusters based on co-expression of ligands in sending cells and cognate receptors in receiving cells, generating directional communication networks that provide mechanistic hypotheses for intercellular regulation. In LN, several disease-relevant communication axes have been identified through this approach.

Analysis of AMP LN biopsy data using NicheNet (99) identified a macrophage-to-TEC communication network centered on CSF1-CSF1R, TNF-TNFR1, and IL-1β-IL-1R1 axes, predicting that macrophage-derived signals drive IFN response gene upregulation and apoptotic pathway activation in proximal TECs. In a separate study applying CellChat (100) to LN kidney scRNA-seq data, SPP1 (osteopontin) signaling from macrophages to fibroblasts was identified as a major pro-fibrotic communication pathway in Class IV biopsies with high chronicity index, consistent with reports of elevated urinary osteopontin in LN patients with progressive renal dysfunction. These computationally inferred communication networks provide directional hypotheses that require experimental validation, through co-culture assays, conditional knockout models, or spatial proximity confirmation, before serving as the basis for therapeutic targeting.

### 5.8 Technical limitations and methodological considerations for single-cell and multi-omics studies in LN

While single-cell and spatial multi-omics technologies have transformed the understanding of LN, several methodological limitations must be acknowledged. Tissue dissociation protocols required for scRNA-seq introduce biases: enzymatic digestion preferentially recovers certain cell types while underrepresenting others (e.g., fibroblasts and mesangial cells may be overrepresented relative to fragile podocytes), and the dissociation process itself can induce transcriptional stress artifacts that confound the identification of disease-specific gene expression programs. Strategies such as cold-active protease protocols and computational removal of dissociation-induced genes partially mitigate these effects but do not eliminate them entirely.

Batch effects, systematic technical variation between samples processed on different days, at different sites, or with different reagent lots, remain a significant challenge in multi-sample single-cell studies. Although computational integration methods (Harmony, Scanorama, scVI) can reduce batch effects, aggressive correction risks removing genuine biological variation. Standardization of sample collection, processing, and bioinformatic pipelines across centers remains incomplete, limiting the comparability of datasets generated by different groups.

Compatibility with formalin-fixed paraffin-embedded (FFPE) tissue is an important practical consideration, as most clinical kidney biopsies are processed as FFPE rather than fresh-frozen material. While spatial transcriptomic platforms (Visium FFPE, Xenium, CosMx SMI) are increasingly optimized for FFPE, single-cell dissociation-based approaches require fresh or cryopreserved tissue, limiting their applicability to prospectively collected biopsy material. Furthermore, most studies to date have been cross-sectional with small sample sizes (typically 20–50 biopsies), enriched for patients of East Asian or European ancestry, and performed at a limited number of academic centers. Longitudinal single-cell studies tracking molecular changes across treatment and disease phases are essential but logistically challenging due to the invasive nature of repeat biopsy. Finally, the cost and technical complexity of high-resolution platforms currently restrict their clinical deployment; development of simplified, cost-effective surrogate assays that approximate single-cell resolution information is necessary for broader implementation.

A particularly important and underappreciated limitation concerns the demographic representativeness of the datasets that currently dominate the LN single-cell literature. The AMP RA/SLE Network, which has generated the most comprehensive and widely cited single-cell atlas of LN, enrolled patients predominantly from North American academic medical centers, with a cohort composition that reflects the demographics of those institutions (8). While the AMP consortium includes patients of African American and Hispanic ancestry, the representation of East Asian populations, who constitute a substantial proportion of global LN patients, remains limited. This is a consequential gap for several reasons. First, LN epidemiology differs significantly between populations: East Asian patients with SLE have a higher incidence and prevalence of renal involvement than European-ancestry patients, and the distribution of ISN/RPS histological classes may differ, with some studies reporting higher rates of Class V membranous LN in certain Asian cohorts (3, 101). Second, genetic risk architecture for SLE and LN varies across ancestries: GWAS studies have identified ancestry-specific risk loci and allele frequency differences at shared loci (such as TNFSF4, STAT4, and BLK) that may influence immune cell gene expression programs in ways not captured by cohorts of predominantly European or African American ancestry (102). Third, treatment practices and background immunosuppressive regimens differ between regions, for example, multi-target therapy combining mycophenolate with tacrolimus has been widely adopted in China and other East Asian countries but is less common in North American protocols (103), and these therapeutic differences may influence the immune landscape captured at biopsy. Consequently, cell type proportions, transcriptional programs, and ligand-receptor communication networks identified in AMP-derived data cannot be assumed to be directly generalizable to East Asian LN populations without independent validation. Emerging single-cell studies from Chinese, Japanese, and Korean cohorts are beginning to address this gap, but most remain limited in sample size and have not yet been systematically compared with the AMP reference atlas. International collaborative efforts that explicitly incorporate globally diverse cohorts, particularly from the high-burden regions of East and Southeast Asia, are essential to ensure that molecular stratification frameworks and candidate biomarkers are broadly applicable rather than population-specific.

An additional dimension of demographic representativeness that warrants attention is biological sex. Given that SLE disproportionately affects women (female-to-male ratio approximately 9:1), and that LN occurs in a higher proportion of male SLE patients despite the overall female predominance, sex-specific differences in intrarenal immune cell composition and molecular pathogenic programs represent a critically underexplored area. Current multi-omic datasets, while predominantly composed of female patients reflecting the epidemiology of SLE, have not been systematically analyzed for sex-stratified differences in immune cell proportions, transcriptional programs, or treatment responses. Emerging evidence from other autoimmune conditions suggests that sex hormones influence immune cell polarization, IFN pathway activity, and B cell hyperactivation, raising the possibility that molecular endotype distributions may differ between male and female LN patients. Future multi-omic studies should incorporate sex as an analytical variable to determine whether sex-specific molecular signatures exist and whether they carry implications for therapeutic stratification.

## 6 The evolving biomarker landscape in lupus nephritis: conventional serology through multiplexed molecular panels

Reliable biomarkers are central to the clinical management of LN. An ideal LN biomarker should accurately reflect current renal inflammatory activity, predict flares before clinically apparent relapse, distinguish LN from other causes of renal dysfunction, correlate with histological findings to potentially reduce biopsy frequency, and respond to treatment in a manner that predicts long-term renal outcomes. No single currently available biomarker fulfills all these criteria, but a growing repertoire of molecular candidates, spanning serum, urine, and tissue compartments, is approaching clinical validation.

To organize the biomarker candidates reviewed below, we propose a tiered classification analogous to the technology readiness levels used in engineering and drug development. At Tier 1 (discovery), a biomarker has been identified through hypothesis-generating omics analysis, typically in a single cohort; most single-cell-derived LN biomarkers currently reside at this level. At Tier 2 (technical validation), the biomarker has been measured reliably across independent laboratories using standardized assays, with acceptable coefficients of variation; urinary MCP-1/CCL2 and CXCL10 have reached this stage. At Tier 3 (clinical validation), the biomarker has demonstrated consistent associations with clinically meaningful endpoints in adequately powered, prospective, multi-ethnic cohorts, a threshold that, to our knowledge, no LN-specific molecular biomarker has yet fully crossed, though urinary CXCL10 and sCD163 are approaching it (53, 70). At Tier 4 (clinical utility), the biomarker has been shown in interventional studies to improve patient outcomes when used to guide clinical decisions compared with standard monitoring, a level not yet achieved for any LN biomarker. This classification is not intended to diminish the importance of discovery-phase work, but rather to provide a realistic calibration of how far the field must travel from its current position to clinical implementation, and to highlight the specific types of studies, particularly prospective interventional trials with biomarker-guided treatment arms, that are most urgently needed.

To provide explicit guidance, we assign the major biomarker candidates discussed in this review to their current tier positions: Tier 1 (discovery): urinary IL-16, urinary EV-derived proteomic panels, cfDNA fragmentomic profiles, urinary miR-29c, and tissue-derived TREM2+ macrophage signatures. Tier 2 (technical validation): urinary MCP-1/CCL2, urinary CXCL10, urinary CXCL9, urinary sCD163, serum BAFF levels, and the IFN-TEC deconvolution metric. Tier 3 (approaching clinical validation): urinary CXCL10 (the most advanced, with consistent associations across multiple independent cohorts) and urinary sCD163 (with emerging multi-center data). Tier 4 (clinical utility): no LN-specific molecular biomarker has yet reached this level. Anti-dsDNA antibodies and serum complement (C3/C4), while clinically established, are systemic rather than tissue-specific markers and were not designed within the molecular endotyping framework.

### 6.1 Traditional serological and urinalysis markers

Anti-double-stranded DNA (anti-dsDNA) antibodies and complement consumption (manifesting as reduced serum C3 and C4 concentrations) have served as conventional serological indicators of SLE disease activity and LN flare risk for decades. Anti-dsDNA titers characteristically rise 2–3 months prior to clinical LN flares in a proportion of patients, creating a potential window for pre-emptive treatment intensification, although their sensitivity and specificity are insufficient to serve as sole clinical decision-making tools. Hypocomplementemia accompanies active nephritis in approximately 60–75% of cases and is incorporated into several composite disease activity scoring systems; nevertheless, a subset of patients with histologically confirmed Class III/IV LN maintain normal serum complement levels, which limits the negative predictive value of this parameter. Proteinuria quantification—assessed via spot urine protein-to-creatinine ratio (uPCR) or 24-hour urine collection—remains the principal monitoring parameter for treatment response evaluation, with target uPCR below 0.5 g/g (or complete renal response defined as uPCR < 0.2–0.3 g/g with stable serum creatinine) serving as the benchmark for response assessment in contemporary clinical trials (104).

Urinary red cell casts, when present, are highly specific for glomerulonephritis and indicate active glomerular inflammation. Active urinary sediment, defined by the combination of haematuria, pyuria, and cellular casts, correlates with histological Class III/IV LN but is absent in 20–30% of patients with biopsy-confirmed active nephritis, underscoring the need for more sensitive molecular indicators. Serum creatinine and estimated glomerular filtration rate (eGFR) reflect cumulative renal function and chronicity rather than acute inflammatory activity; they are relatively insensitive indicators of early or moderate LN flares and may not decrease substantially until substantial nephron loss has occurred.

### 6.2 Urinary chemokine and cytokine molecular markers

Urinary chemokines synthesized within the inflamed kidney provide the analytical advantage of reflecting local renal immune processes rather than systemic inflammatory activity. MCP-1/CCL2 ranks among the most extensively validated urinary molecular indicators in LN, demonstrating elevated concentrations during active disease compared with quiescent phases across multiple independent validation cohorts, reproducible correlation with histological activity indices, and measurable decline with effective immunosuppressive treatment (69). Urinary CXCL10 (IP-10), a chemokine transcriptionally induced by IFN-γ signaling in renal tubular and mesangial cells, has shown consistent correlation with kidney histological activity and reliably discriminates active LN from alternative causes of proteinuria across multiple independent cohorts (53). Urinary CXCL9 (MIG), an additional CXCR3-binding chemokine produced under IFN-γ stimulation, demonstrates comparable discriminative performance and has been proposed as complementary to CXCL10, given their partially non-overlapping expression patterns across different nephron segments (105).

Soluble CD163 (sCD163), a scavenger receptor shed from activated M2 macrophages, is detectable in urine and has been identified as a biomarker of macrophage-driven renal inflammation. Urinary sCD163 levels are markedly elevated in active LN, correlate with interstitial macrophage infiltration on biopsy, and predict renal relapse in patients achieving clinical remission (70). More recently, Fava et al. integrated urine proteomics with renal single-cell transcriptomics and identified interleukin-16 (IL-16) as a novel urinary biomarker candidate in LN; urinary IL-16 levels correlated with intrarenal lymphocyte infiltration and distinguished active LN from inactive disease, adding to the expanding repertoire of non-invasive molecular indicators (106).

Emerging evidence suggests that multi-analyte urinary panels combining CXCL10, sCD163, IL-16, and tubular activation markers such as ALCAM may outperform single-biomarker approaches and conventional serology and urinalysis for distinguishing active from inactive LN, though large-scale multicenter validation remains ongoing. Prospective validation of such multi-analyte panels in ethnically diverse cohorts and across different treatment regimens is ongoing.

### 6.3 Tissue-derived molecular biomarker candidates

Integration of single-cell transcriptomic data from LN biopsies with clinical outcomes has identified candidate tissue biomarkers with prognostic relevance beyond conventional histological classification. The IFN-stimulated tubular epithelial cell (IFN-TEC) cluster identified in the AMP Phase 1 study, characterized by expression of IFIT1, MX1, OAS1, and CXCL10, correlates with histological activity score and is more abundant in Class III/IV than Class II biopsies (8). Quantification of IFN-TEC abundance by deconvolution of bulk RNA-seq from biopsy material, or by immunohistochemical surrogate markers, may provide a reproducible tissue activity metric applicable without requiring single-cell sequencing infrastructure.

The TREM2+ pro-fibrotic macrophage cluster, enriched in biopsies with higher chronicity index, represents a potential tissue biomarker of ongoing fibrogenesis that is not captured by conventional chronicity scoring. TREM2 protein can be quantified by immunohistochemistry on standard biopsy sections, and urinary TREM2+ macrophage-derived exosomes are detectable in urine, suggesting a non-invasive correlate of the intrarenal fibrotic macrophage program. Spatial transcriptomic characterization of the tubulo-immune niche, the co-localization of IFN-stimulated TECs and inflammatory macrophages identified by Visium mapping, may ultimately provide a spatially resolved activity metric that predicts response to IFN pathway-targeting therapies.

Molecular stratification of LN patients based on biopsy transcriptomic profiles has identified at least two reproducible disease endotypes in independent cohorts: a high-IFN/high-lymphocyte endotype enriched in Class III/IV LN with prominent tubulointerstitial inflammation, and a low-IFN/myeloid-dominant endotype more prevalent in Class V (membranous) LN and associated with greater proteinuria but less cellular infiltrate. These endotypes show differential responses to standard induction therapy in retrospective analyses, suggesting that transcriptomic stratification could ultimately guide treatment selection, though clinical validation remains necessary before implementation (see Section 7.8).

However, the robustness of these endotype classifications warrants careful scrutiny. Several concerns merit explicit discussion. First, the definition of “endotype” in LN lacks a standardized framework: different studies have used varying numbers of input genes, clustering algorithms—for example, Leiden community detection in the AMP Phase 2 analyses (8, 88), k-means partitioning in earlier transcriptomic subtyping efforts (9), and hierarchical clustering for gene co-expression module identification (89)—normalization methods, and cohort compositions to arrive at endotype designations, and it has not been systematically evaluated whether these different analytical approaches assign the same patients to the same endotype categories. What one study labels an “IFN-high” endotype may not correspond precisely to the similarly named category in another study, because the gene sets defining the IFN signature, the threshold for “high” versus “low” expression, and the background population used for comparison all differ. Second, most endotype studies have been performed on cross-sectional biopsy material; whether a patient’s endotype is a stable molecular trait or a transient state that fluctuates with disease phase, treatment exposure, and intercurrent events remains unknown. If endotypes are dynamic, their clinical utility for guiding long-term treatment selection is substantially diminished. Third, there is a risk of circularity in endotype validation: when the same cohort is used for both endotype discovery and outcome association, apparent prognostic significance may reflect overfitting rather than genuine biological stratification. Independent validation in entirely separate, prospectively enrolled cohorts, ideally across multiple ethnic backgrounds and treatment protocols, is the minimum standard required before endotype classifications can be considered clinically established, and this standard has not yet been met for any proposed LN endotype scheme.

### 6.4 Cell-free DNA and epigenetic circulating biomarkers

Cell-free DNA (cfDNA) in plasma originates from dying cells and has been investigated as a disease activity biomarker in LN. Total cfDNA levels are elevated in active SLE compared with inactive disease, and mitochondrial cfDNA, which is a potent IFN-I inducer, is disproportionately elevated in patients with active nephritis. Chan et al. demonstrated that plasma DNA from active SLE patients exhibits aberrant fragment-size profiles, hypomethylation, and altered genomic representations correlating with disease activity, providing proof of concept that cfDNA fragmentomics may serve as a non-invasive readout of SLE/LN activity (107). Cell-type-specific methylation patterns in cfDNA (applying methods first demonstrated for β cells and oligodendrocytes by Lehmann-Werman et al. (108)) may eventually enable discrimination of immune cell-derived versus parenchymal cell-derived DNA, potentially distinguishing systemic from intrarenal drivers of disease activity.

## 7 Mechanism-directed therapeutic strategies mapped to disease architecture

The mechanistic insights generated by single-cell, spatial, and multi-omics studies of LN are beginning to converge with therapeutic development. This section reviews treatment advances and emerging strategies, with emphasis on those directly informed by molecular profiling data. Standard induction and maintenance regimens, mycophenolate mofetil, cyclophosphamide, glucocorticoids, hydroxychloroquine, remain the backbone of LN therapy and are discussed briefly as context; the focus here is on targeted agents with mechanistic rationale grounded in the pathobiology described in preceding sections.

**Table 3** summarizes the emerging therapeutic agents discussed in this section, including mechanism of action, target pathway, key clinical trial data, regulatory status, and their position within the convergent-to-divergent therapeutic framework. This table is intended to provide a quick-reference guide complementing the detailed narrative discussion of each agent in the subsections below (109, 110).

**Table 3:** Emerging therapeutic agents in lupus nephritis.

Therapeutic agent	Mechanism	Target pathway	Conv./div.	Key trial	Phase	Key result	Status	Ref
Voclosporin	CNI; calcineurin-NFAT inhibition + podocyte stabilization	T cell activation; podocyte cytoskeleton	Convergent + Divergent (podocyte)	AURORA	III	CRR 40.8% vs 22.5% at 52 wk (p<0.001)	FDA/EMA approved for LN	(104)
Belimumab	Anti-BAFF mAb; inhibits B cell survival	B cell/plasma cell pathway	Divergent (B cell/TLS)	BLISS-LN	III	PERR 43% vs 32% at wk 101 (p=0.03)	FDA/EMA approved for LN	(66)
Obinutuzumab	Anti-CD20 mAb (enhanced ADCC); B cell depletion	B cell depletion; DN2 B cells	Divergent (B cell/TLS)	NOBILITY; REGENCY	II; III	CRR 41% vs 23% (wk 101); REGENCY: CRR 46.4% vs 33.1% (wk 76)	Approved (REGENCY)	(113, 114)
Anifrolumab	Anti-IFNAR1 mAb; blocks all type I IFN signaling	Type I IFN pathway	Divergent (IFN-driven)	TULIP-LN; IRIS (ongoing)	II; III	CRR 45.5% vs 31.1% (IR arm, phase II)	Phase III ongoing	(115, 116)
Baricitinib	JAK1/2 inhibitor; blocks IFN, IL-6, IL-2 signaling	JAK-STAT axis (multi-cytokine)	Convergent (JAK node)	SLE-BRAVE-I/II	III	BRAVE-I: SRI-4 57% vs 46% (p=0.016); BRAVE-II: NS	Investigational in LN	(117, 118)
Avacopan	C5aR1 antagonist; blocks C5a-mediated inflammation	Complement (C5a effector arm)	Convergent (complement)	Under evaluation in LN	Early	Approved for ANCA vasculitis; LN data pending	Investigational in LN	(119)
Telitacicept	Dual BAFF/APRIL blockade (TACI-Fc fusion)	B cell and plasma cell survival	Divergent (B cell/plasma cell)	Phase 2b in SLE	IIb	SRI-4 response improved vs placebo in SLE	Investigational	(81)
Low-dose IL-2	Selective Treg expansion via IL-2R signaling	Treg/Teff balance	Convergent (immune regulation)	RCT in SLE; NCT04779307 (LN)	II	Treg expansion; renal activity reduction signals	Phase II ongoing in LN	(61, 122)
Anti-CD19 CAR-T	Deep B cell + plasmablast depletion via engineered T cells	B cell lineage (CD19+)	Divergent (deep B cell reset)	Erlangen series; expanded cohorts	I/II	Drug-free remission in refractory SLE/LN (n=5-15)	Investigational; specialized centers	(123–125)

The SLE-BRAVE-I and SLE-BRAVE-II trials for baricitinib were conducted in a general SLE population rather than a dedicated LN cohort. Results listed under the baricitinib entry reflect SLE-wide efficacy data and should not be directly extrapolated to LN-specific therapeutic outcomes. Dedicated LN trials of JAK inhibitors are ongoing (see Section 7.4).

To organize the therapeutic landscape within the convergent-to-divergent framework that is central to this review, we distinguish between two categories of interventions. Convergent-targeting therapies, including mycophenolate mofetil, cyclophosphamide, and calcineurin inhibitors, act on shared upstream pathogenic mechanisms (immune complex-mediated inflammation, broad lymphocyte activation) and remain the backbone of induction and maintenance treatment. Divergent-targeting therapies, including belimumab (B cell/BAFF pathway), anifrolumab (type I IFN pathway), baricitinib (JAK-STAT signaling), avacopan (complement C5a receptor), and CAR-T cell approaches (deep B cell depletion), are designed to address specific molecular branches that dominate in subsets of patients. This framing helps explain why broad immunosuppression achieves remission in many patients (those whose disease is driven primarily by convergent mechanisms) while failing in others (those whose renal injury is sustained by a dominant divergent pathway that is not adequately targeted). As each therapeutic agent is discussed below, we indicate where in the convergent-to-divergent model it acts and which patient endotype(s) are most likely to benefit (see **Figure 3**) (8, 15, 16).

### 7.1 Standard-of-care immunosuppression: induction and maintenance protocols

Contemporary evidence-based induction treatment for Class III/IV LN comprises mycophenolate mofetil (MMF, 2–3 g/day) or low-dose intravenous cyclophosphamide (administered per the Euro-Lupus protocol at 500 mg fortnightly for six doses) in combination with glucocorticoids. Both approaches achieve complete renal response in approximately 20–30% of patients at six months and partial response in an additional 30–40%, leaving a substantial proportion with therapeutically inadequate outcomes (111). Maintenance immunosuppression with MMF or azathioprine, combined with continuous hydroxychloroquine, mitigates relapse risk and is typically sustained for a minimum of three years following induction (112). Glucocorticoid dose minimization strategies, supported by outcome data from the BLISS-LN and AURORA trials, are being increasingly adopted to attenuate long-term metabolic, cardiovascular, and infectious adverse effects (67).

These regimens are codified within international guideline frameworks. The 2019 EULAR/ERA-EDTA recommendations, updated in 2020, formalised MMF as the preferred induction agent for most patients with Class III/IV LN, with low-dose intravenous cyclophosphamide (Euro-Lupus protocol) as an equivalent alternative and high-dose cyclophosphamide reserved for severe or life-threatening disease (113). The same guidelines designated hydroxychloroquine as background therapy for all LN patients in view of its nephroprotective and immunomodulatory properties, independent of disease class. Glucocorticoid doses are recommended to be tapered to no more than 7.5 mg/day prednisolone equivalent within the first three to six months of induction (113). Treatment response targets have been progressively refined: complete renal response, defined as urine protein-to-creatinine ratio below 0.5 g/g with stable renal function, at 12 months is the primary benchmark in clinical trials, while a stricter target below 0.25–0.3 g/g at 24 months is associated with superior long-term renal outcomes and is endorsed as the preferred endpoint in current trials (104). Partial response (uPCR reduction exceeding 50% from baseline with stabilisation or improvement of eGFR) at six months identifies patients at high risk of long-term progression and should prompt consideration of early treatment intensification or re-biopsy. Notably, current guidelines emphasise that newly approved targeted agents, including voclosporin and belimumab, are to be added to, rather than substituted for, background MMF and hydroxychloroquine, reinforcing the combination immunosuppression framework as the contemporary standard of care (104, 113).

### 7.2 Voclosporin: a dual-mechanism calcineurin inhibitor

Voclosporin, a structurally modified calcineurin inhibitor (CNI) engineered for more predictable pharmacokinetic behavior than tacrolimus or cyclosporine, has demonstrated robust efficacy as add-on therapy to MMF and glucocorticoids in the phase III AURORA trial. The voclosporin-MMF combination yielded complete renal response in 40.8% of patients at 52 weeks versus 22.5% in the MMF-alone control arm (104), constituting a clinically meaningful improvement in induction response rates. The dual mechanism of action encompasses T cell suppression through calcineurin-NFAT pathway inhibition alongside direct podocyte cytoprotection via actin cytoskeletal stabilization. Whether pre-treatment molecular profiling can prospectively identify the patients most likely to derive benefit from voclosporin remains an unresolved question being addressed by ongoing biomarker sub-studies.

### 7.3 B cell-directed therapy: belimumab, obinutuzumab, and ectopic lymphoid disruption

Belimumab, a fully human monoclonal antibody directed against soluble BAFF, became the first biologic agent to achieve regulatory approval specifically for active LN, supported by the BLISS-LN phase III trial that demonstrated superior primary efficacy renal response at week 101 relative to standard of care (43% vs 32%, p , 0.03) (67). At the mechanistic level, belimumab depletes circulating plasmablast and transitional B cell populations, lowers anti-dsDNA antibody titers, and reduces complement consumption; *post-hoc* biomarker analysis of the BLISS-LN dataset revealed that patients with the highest baseline plasmablast frequencies and serum BAFF concentrations derived the greatest absolute clinical benefit, providing a preliminary molecular predictor of treatment response. Obinutuzumab, a glycoengineered type II anti-CD20 monoclonal antibody with enhanced antibody-dependent cellular cytotoxicity (ADCC) activity, was developed partly in response to rituximab’s failure to demonstrate superiority over placebo in the phase III LUNAR trial (114). Obinutuzumab demonstrated improvement in complete renal response rates in the phase II NOBILITY trial (CRR 35% vs 23%, p , 0.115 at the primary week 52 endpoint; by week 101, CRR reached 41% vs 23%, p , 0.026) (115), with sustained peripheral B cell depletion maintained throughout the treatment period. Single-cell molecular profiling of peripheral blood B cell populations from obinutuzumab-treated patients revealed preferential depletion of the DN2 autoimmune-associated B cell subset, suggesting that this pathogenic population—originally identified through scRNA-seq studies—may represent a particularly relevant cellular target for anti-CD20 therapy in LN. These observations received confirmatory support from the phase III REGENCY trial, in which obinutuzumab added to standard of care achieved a complete renal response rate of 46.4% versus 33.1% for placebo at week 76, establishing obinutuzumab as an effective addition to the LN therapeutic armamentarium (116).

### 7.4 Interferon pathway blockade: anifrolumab and JAK inhibitor approaches

Anifrolumab, a monoclonal antibody directed against the type I IFN receptor subunit IFNAR1, inhibits signaling from all IFN-I subtypes and has demonstrated efficacy in reducing SLE disease activity in the phase III TULIP-2 trial (117) (which achieved its primary BICLA endpoint), whereas the TULIP-1 trial did not reach its primary SRI-4 endpoint despite numerical trends favoring anifrolumab. Notably, the two trials employed different primary endpoints—BICLA for TULIP-2 and SRI-4 for TULIP-1—reflecting evolving views on optimal outcome measurement in SLE; the discordant results may therefore reflect differences in endpoint sensitivity to anifrolumab’s mechanism of action rather than a true inconsistency in drug efficacy. In a dedicated phase II trial evaluating anifrolumab in active LN (TULIP-LN), addition to standard of care yielded numerically higher complete renal response rates at week 52 versus placebo in the intensified regimen arm (45.5% vs 31.1%), although this three-arm phase II trial was not statistically powered for formal hypothesis testing (118). *Post-hoc* biomarker analysis demonstrated that therapeutic benefit was concentrated among patients with elevated baseline IFN gene signature scores, consistent with the mechanistic rationale for selective IFN pathway inhibition (118). A confirmatory phase III trial in LN (IRIS) is actively recruiting, incorporating IFN gene signature score as both a randomization stratification variable and an exploratory biomarker endpoint.

JAK inhibitors provide an alternative approach to IFN pathway blockade by inhibiting JAK1/TYK2-mediated IFNAR signaling downstream of the receptor. Baricitinib (JAK1/2 inhibitor) showed efficacy in the phase III SLE-BRAVE-I trial (SRI-4 response 57% vs 46% placebo, p , 0.0160) but failed to replicate this in SLE-BRAVE-II (SRI-4 47% vs 46%, not significant) (119, 120), highlighting the heterogeneity of treatment response in SLE. Upadacitinib (selective JAK1 inhibitor) is under evaluation in a dedicated LN trial. Notably, JAK inhibitors simultaneously suppress IL-6, IL-2, and multiple additional cytokine pathways, potentially providing broader immunomodulatory effects than selective IFN-I blockade at the cost of a broader adverse effect profile.

### 7.5 Complement pathway modulation: broad-spectrum targeting with pathway-specific implications

Given the established role of complement activation in glomerular injury, complement-targeted therapy has been a longstanding area of interest in LN. Avacopan, a selective C5a receptor (C5aR1) antagonist approved for ANCA-associated vasculitis (121), is under evaluation in LN based on evidence of C5a-mediated neutrophil and macrophage recruitment in glomerulonephritis. C1s inhibition with sutimlimab, approved for cold agglutinin disease, blocks the classical complement pathway at its initiating step and is being evaluated in immune complex-mediated nephritis, including a pilot study in LN. Early-phase trials of anti-C5 therapy (eculizumab) in LN have shown inconsistent results, possibly reflecting the fact that complement activation occurs upstream of C5 in some LN patients via the lectin or alternative pathways (122).

### 7.6 Co-stimulatory pathway blockade and T cell-directed interventions

Aberrant T cell activation and Tfh-B cell collaboration are rational targets for co-stimulation blockade in LN. Abatacept (CTLA4-Ig), which blocks CD28-CD80/86 co-stimulation, showed no benefit over placebo in a phase II randomized trial in proliferative LN when added to standard immunosuppression (123), possibly due to incomplete suppression of the co-stimulatory signal in the presence of high BAFF levels that rescue B cells independently of T cell help. However, a *post-hoc* analysis of a subset enriched for anti-dsDNA positivity and hypocomplementemia suggested benefit in this serologically active subgroup, and a dedicated trial in this molecularly defined population is being considered.

Low-dose IL-2 therapy, as discussed in Section 3.1, selectively expands Tregs without globally immunosuppressing effector T cells and has shown signals of renal activity reduction in pilot studies. A prior randomised trial demonstrated the efficacy and safety of low-dose IL-2 in SLE, including signals of renal activity reduction (124). A dedicated phase II trial specifically in LN is ongoing in China (NCT04779307), with urinary CXCL10 and urinary sediment cell transcriptomics as exploratory biomarker endpoints. Dapirolizumab pegol, an anti-CD40L PEGylated Fab targeting the CD40-CD40L co-stimulatory axis, critical for T cell help to B cells and macrophage activation, is under evaluation in SLE following encouraging phase II results, though dedicated LN data are limited.

### 7.7 Comprehensive immune reconstitution: CAR-T cell therapy and plasma cell targeting

Chimeric antigen receptor T (CAR-T) cell therapy directed against CD19 has recently entered clinical evaluation in severe, treatment-refractory SLE. An initial case series of five patients with refractory SLE—four of whom had active LN as a major disease manifestation—treated with CD19-targeted CAR-T cells at the University of Erlangen-Nuremberg achieved sustained drug-free remission accompanied by normalization of complement levels, anti-dsDNA titers, and urinary sediment analysis over follow-up periods initially extending to eighteen months (125). Importantly, longer-term follow-up data are now available: Müller et al. reported an expanded series of 15 patients with severe autoimmune conditions (encompassing SLE, idiopathic inflammatory myopathy, and systemic sclerosis) treated with CD19 CAR-T cells, with follow-up now extending beyond three years in the earliest-treated patients. These data confirm durable drug-free remissions with sustained seroconversion (anti-dsDNA negativity), normalized complement levels, and immune reconstitution characterized by a non-autoreactive B cell repertoire (126). Notably, the durability of response appears encouraging, with no disease relapses reported in the SLE patients during extended follow-up, although the small sample size and single-center nature of these observations warrant caution in generalization. Wang et al. additionally reported outcomes with a BCMA-CD19 compound CAR-T cell construct directed against both CD19+ B cells and BCMA+ plasma cells in SLE patients, documenting rapid and sustained autoantibody titer reduction with concurrent improvement in renal functional parameters, suggesting that dual-target CAR-T strategies may achieve more thorough elimination of the autoreactive B cell lineage (127). The mechanism of CAR-T efficacy involves comprehensive depletion of CD19+ B cells and their developmental precursors including autoreactive plasmablasts, with subsequent immune system reconstitution from naive B cell precursors exhibiting reduced autoreactive potential. These observations are noteworthy for the depth and durability of clinical responses achieved, though they require validation in larger, controlled patient cohorts with extended follow-up, and the manufacturing and administration logistics of CAR-T therapy currently restrict this approach to specialized academic centers.

CD38-targeted therapy with daratumumab, a monoclonal antibody approved for multiple myeloma, depletes CD38+ plasma cells, which are responsible for autoantibody production and are relatively resistant to conventional B cell-depleting agents that target CD20 (a marker largely absent on terminally differentiated plasma cells). Case reports with detailed immunological analysis in refractory SLE have documented rapid reduction in anti-dsDNA titers and improvement in renal parameters with daratumumab, with a manageable safety profile (128). Inclusion of plasma cell-depleting strategies alongside conventional B cell depletion may address the longstanding problem of autoantibody persistence following rituximab or obinutuzumab therapy.

### 7.8 Translating molecular disease subtypes into biomarker-stratified trial designs

The overarching objective of molecular profiling in LN is to enable individualized therapeutic decision-making—matching each patient to the treatment strategy most likely to produce a favorable response based on their specific molecular disease profile. The conceptual paradigm parallels the precision oncology model, in which tumor molecular characterization directly guides targeted agent selection. In LN, three broad molecular disease subtypes are crystallizing from multi-omics datasets: an IFN-high/lymphocyte-predominant subtype predicted to respond favorably to IFN pathway blockade and B cell-directed therapy; a myeloid/macrophage-predominant subtype potentially amenable to JAK pathway inhibition or CSF1R-targeted approaches; and a fibrotic/TGF-β-predominant subtype in which anti-fibrotic rather than conventional anti-inflammatory interventions may warrant prioritization.

However, a critical examination of the molecular endotyping concept in LN reveals several unresolved challenges. First, the number, boundaries, and reproducibility of proposed endotypes are not yet settled. Second, it remains unclear whether an individual patient’s endotype is stable over time or shifts with treatment and disease evolution. Third, while the multi-omic evidence reviewed here provides a compelling scientific foundation for endotype-informed therapeutic selection, the ultimate clinical validation of this approach requires prospective, endotype-stratified clinical trials that directly compare biomarker-guided treatment selection with current standard-of-care regimens. Such trials, which should be designed as the field’s highest priority, will determine whether the substantial molecular heterogeneity documented across studies translates into clinically meaningful differences in treatment response, as the convergent-to-divergent framework predicts. Importantly, the available evidence strongly favors the hypothesis that distinct molecular endotypes drive divergent clinical trajectories and will ultimately require tailored interventions; the endotype-stratified trial is needed not to establish whether heterogeneity exists, but to quantify the clinical benefit of matching therapy to molecular profile (8, 88, 129).

Translation of this conceptual framework into routine clinical practice necessitates prospective randomized trials incorporating biomarker-stratified treatment allocation arms—an approach currently being piloted within several multinational academic research consortia. Critical methodological hurdles include the requirement for rapid-turnaround molecular assay processing to inform time-sensitive induction therapy decisions (biopsy specimens must be processed within days rather than weeks), the substantial cost and infrastructure demands of deploying single-cell profiling platforms in clinical laboratory settings, and the regulatory framework required for approval of biomarker-guided treatment allocation strategies. In the near term, simplified surrogate biomarker panels—such as urinary CXCL10 as a proxy for IFN-dominant disease, urinary sCD163 for macrophage-predominant activity, and circulating plasmablast enumeration for B cell-driven pathology—may offer clinically actionable molecular indicators that approximate the information content of comprehensive transcriptomic profiling at substantially reduced cost and technical complexity.

## 8 Integrative synthesis and priorities for clinical translation

Notwithstanding substantial recent therapeutic advances—including the regulatory approvals of voclosporin and belimumab and promising clinical data for obinutuzumab, anifrolumab, and chimeric antigen receptor T cell approaches—LN continues to rank among the most therapeutically challenging autoimmune organ manifestations. A substantial proportion of patients demonstrate suboptimal treatment responses, experience disease relapse following initial remission, or suffer progressive and irreversible renal function decline, highlighting the inherent limitations of existing empirically derived treatment protocols.

The most impactful mechanistic insights have emerged from single-cell and spatially resolved transcriptomic investigations, which have constructed a cellular atlas of LN at previously unattainable molecular resolution. Major contributions include: identification of disease-linked immune cell states—IFN-activated TECs, inflammatory bone marrow-derived macrophages, DN2 B cells, CXCL13-expressing Tfh-like cells—that demonstrate reproducible associations with histological activity and patient outcomes; characterization of intrarenal intercellular signaling networks including the macrophage-TEC inflammatory co-activation circuit; mapping of organized tertiary lymphoid structures (TLS) sustained by CXCL13-producing stromal cells; and delineation of at least two reproducible molecular disease subtypes with distinct therapeutic implications.

Drawing together these findings, we revisit the shared-to-branching pathogenic framework articulated in Section 1. The accumulated evidence now convincingly demonstrates that LN, while originating from common upstream immunological triggers, branches into molecularly distinct tissue-level disease trajectories—a phenomenon with direct clinical consequences, as it likely accounts for the heterogeneous treatment responses consistently observed across randomized clinical trials. The essential next question is whether prospective identification of these molecular trajectories and matching individual patients to mechanism-appropriate therapies yields measurably superior outcomes relative to current uniform treatment approaches. This is fundamentally an empirical question that cannot be definitively answered through additional observational molecular profiling studies. We therefore advocate that the field’s strategic priorities should pivot from further discovery-phase molecular characterization—an endeavor now yielding diminishing incremental returns—toward the more challenging but clinically decisive work of prospective validation: standardizing rapid-turnaround molecular diagnostic assays, designing subtype-stratified clinical trials, and demonstrating the clinical utility of molecularly guided treatment allocation in ethnically diverse, globally representative patient populations.

Advancement in non-invasive biomarker development has been considerable. Urinary CXCL10, sCD163, and multiplexed analyte panels are approaching analytically validated thresholds for clinical-grade monitoring of renal inflammatory activity, and urine-based molecular profiling approaches—encompassing urinary single-cell transcriptomics, extracellular vesicle proteomics, and cell-free DNA fragmentomic analysis—have established initial proof of concept for detecting intrarenal molecular events without the need for tissue biopsy. Should prospective validation studies confirm these findings, such tools could fundamentally transform the LN monitoring paradigm, enabling substantially more frequent and molecularly informative assessments than serial renal biopsy permits.

Several important limitations and knowledge gaps warrant acknowledgment. The majority of published single-cell and multi-omics investigations to date have been cross-sectional in design, conducted in relatively modest cohorts, and disproportionately enriched for patients of East Asian or European ancestry; longitudinal molecular data from ethnically diverse, globally representative populations remain lacking. Harmonization of specimen collection procedures, tissue processing protocols, and bioinformatic analysis pipelines across participating centers remains incomplete, constraining cross-study dataset comparability. The substantial cost and technical sophistication required by high-resolution profiling platforms currently limit their deployment to specialized academic medical centers; the development of simplified, cost-effective assay formats is a prerequisite for broader clinical adoption.

Looking forward, the intersection of high-resolution molecular profiling capabilities, machine learning and artificial intelligence-driven analytical frameworks (discussed in Section 5.4), and a rapidly expanding portfolio of targeted therapeutic agents collectively positions the field to advance toward authentic precision medicine in LN—although, as emphasized throughout this review, the essential prerequisites of adequate dataset scale, analytical standardization, and rigorous prospective validation must be fulfilled before computational approaches can meaningfully inform clinical management decisions. Encouragingly, recent methodological reviews have highlighted substantial advances in multi-omics integration and prognostic modeling across disease domains, providing an expanding analytical toolkit whose standardization and clinical translation remain active areas of development (130). International collaborative research initiatives, including the AMP consortium and planned expansions to pediatric and ancestrally diverse populations, will be indispensable for achieving the dataset dimensions required for robust molecular stratification. Embedding prospective molecular profiling within the design of ongoing and future clinical trials—serving simultaneously as biomarker measurement endpoints and treatment allocation tools—represents the most direct pathway for translating the molecular discoveries synthesized in this review into tangible clinical benefit for patients with lupus nephritis.
